# Conservation genetics of the capercaillie in Poland - Delineation of conservation units

**DOI:** 10.1371/journal.pone.0174901

**Published:** 2017-04-04

**Authors:** Robert Rutkowski, Dorota Zawadzka, Ewa Suchecka, Dorota Merta

**Affiliations:** 1 Department of Molecular and Biometrical Techniques, Museum and Institute of Zoology Polish Academy of Sciences, Warsaw, Poland; 2 Institute of Forest Science, University of Łódź, Branch in Tomaszów Mazowiecki, Tomaszów Mazowiecki, Poland; 3 Department of Ecology, Wildlife Research and Ecotourism, Pedagogical University of Cracow, Cracow, Poland; Oregon State University, UNITED STATES

## Abstract

The capercaillie (*Tetrao urogallus*) is one of Poland's most endangered bird species, with an estimated population of 380–500 individuals in four isolated areas. To study these natural populations in Poland further, more than 900 non-invasive genetic samples were collected, along with samples from 59 birds representing large, continuous populations in Sweden and Russia; and from two centres in Poland breeding capercaillie. Microsatellite polymorphism at nine loci was then analysed to estimate within-population genetic diversity and genetic differentiation among populations. The results confirmed that isolation of populations and recent decreases in their sizes have reduced genetic diversity among capercaillie in Poland, with all the country’s natural populations found to be experiencing the genetic after-effects of demographic bottlenecks. The results of analyses of genetic differentiation and structure further suggest the presence of a 'lowland' cluster (encompassing birds of the Augustowska and Solska Primaeval Forests in Poland, and of Sweden and Russia), and a Carpathian cluster. Capercaillie from Sweden and Russia are also found to differ markedly. The Polish lowland populations seem more closely related to birds from Scandinavia. Our genetic analysis also indicates that the stocks at breeding centres are of a high genetic diversity effectively reflecting the origins of founder individuals, though identification of ancestry requires further study in the case of some birds.

Overall, the results sustain the conclusion that the Polish populations of capercaillie from the Carpathians and the lowlands should be treated as independent Management Units (MUs). This is to say that the breeding lines associated with these two sources should be maintained separately at breeding centres. The high level of genetic differentiation of birds from the Solska Primaeval Forest suggests that this population should also be assigned the status of independent MU.

## Introduction

The capercaillie (*Tetrao urogallus*, L. 1758) is one of the most intensively studied woodland grouse in Europe, whose range, subspecies, demographic trends, ecology and habitat requirements have all been described in detail, e.g. [1–4]. Currently, continuous populations of the species inhabit boreal forests of the Palearctic. The largest are in Russia (with ca. 2.4 million individuals [5]) and Fennoscandia (ca. 700, 000 birds [3]), though some decrease has been observed recently, even in these regions [6–8]. By contrast, the southwestern part of the range in Western and Central Europe is fragmented—mainly due to the patchy distribution of montane coniferous forests and habitat loss. In this region, the capercaillie is mainly restricted to the Alps, the Pyrenees, the Jura, and the Carpathian and Cantabrian mountains; while many lowland populations have become extinct, and those remaining are small, isolated and threatened [3; 4]. Although according to IUCN the capercaillie is of least concern (LC) at a global level [9], the species is completely protected in 21 European countries, and is Red-Listed in 17 of these [3].

**Fig 1:**
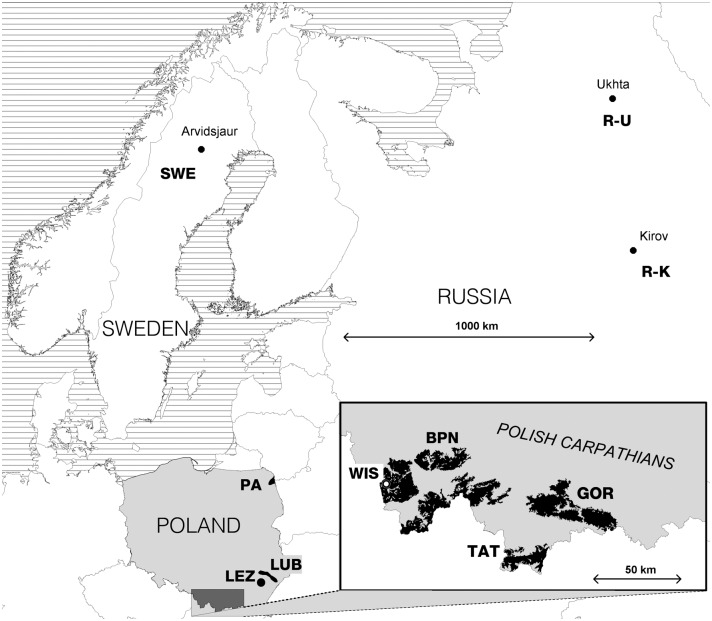
Distribution of sampling populations in northeastern Europe and the Polish Carpathians. PA—Augustowska Primaeval Forest; LUB—Solska Primaeval Forest; GOR—Gorce National Park; TAT—Polish Tatra National Park; BPN—Babia Góra National Park; R-K—Russia, Kirov Oblast; R-U—Russia, Ukhta region in Komi Republic; SWE—Sweden, Norrbotten County; WIS—the breeding centre for capercaillie in Wisła Forest District; LEZ—the breeding centre for capercaillie in Leżajsk Forest District.

The capercaillie is classified as a 'critically endangered' species (CR category) in the Polish Red Book of Animals, and is included in Annex I to the European Union's 'Birds Directive' [10]. While there were still about 2500 capercaillie living in Poland at the beginning of the 20th century [11], a dramatic decline began in the 1970s, when there were an estimated 700–1350 individuals. Populations from Poland's lowland forests (located in northern and central parts) have been disappearing more rapidly than those in mountainous areas (i.e. the south), with the most extreme example being Pomerania (in the northwest), where an estimated population of 150 birds was extirpated in a ten-year period [10]. Poland is now believed to support some 380–500 capercaillie, living within four isolated populations (Fig 1). These birds are in the Polish part of the Western Carpathians, the Primaeval Forests known as Puszcza Solska (LUB in Fig 1) and Puszcza Augustowska (PA in Fig 1), and the Lower Silesian Forest, in which the population is of reintroduced status.

Poland's largest remaining population of the species is that inhabiting the Western Carpathians with 285–325 individuals [12; 13]. The lowland populations of the Solska and Augustowska Primaeval Forests are smaller (70–100 and 30–40 individuals, respectively). The former population seems stable demographically but the latter continues in decline [14– 17], and is now considered highly endangered. Since 2012, efforts have been underway to restore the remnant Augustowska capercaillie population through increasing abundance and genetic diversity [18]. In the Lower Silesian Forest the native population disappeared at the beginning of the 21st century with the last wild birds observed in 2009 [19]. A recovery project was launched in the same year, using captive-reared and wild birds. To date, 151 capercaillie have been reintroduced in this area [20].

Several studies investigating polymorphism in the control region of mitochondrial DNA have shed light on phylogeography of the species and the delineation of its subspecies. The results have indicated no genetic differences between the majority of what were regarded as subspecies, suggesting that Europe has just two genetically identifiable lineages: the southern present in the Cantabrian, Pyrenees, and in the Balkans mountains (mainly the Bulgarian Rhodope and Rila mountains), and the boreal (northern) present in remaining parts of Europe [21–24].

Thus far, the conservation genetics of Polish capercaillie is not well understood. However, preliminary data [25] confirm reduced genetic diversity in birds from isolated strongholds in Poland, while indicating a high level of genetic differentiation among the populations. Since that work was carried out, the natural population of the Lower Silesian Forest has been extirpated, while that in the Augustowska Primaeval Forest, considered the largest in 2005, has declined rapidly [14].

One of the most important steps in planning effective conservation action should be the identification of Conservation Units (CUs), i.e. administrative units that are discrete and biologically relevant [26]. The two commonly recognised CUs are: the Evolutionary Significant Unit (ESU), defined as 'a population or group of populations that merit separate management or priority for conservation because of high distinctiveness (both genetic and ecological)', and the Management Unit (MU), defined as a population significantly divergent from others in terms of allele or haplotype frequencies, regardless of the phylogenetic relationships among those alleles/haplotypes [27; 28].

The primary goal of our investigation was to examine the genetic status of capercaillie in the relevant Conservation Units present in Poland. Additionally, we sought to compare genetic diversity and estimate genetic differentiation among the small and fragmented Polish populations in relation to the large and demographically stable populations present in Russia and Sweden. Taken together, these analyses provide insight allowing important source populations to be identified and their genetic diversity assessed.

## Materials and methods

### Sample collection

Between 2005 and 2014, samples for genetic analysis were collected from eight regions in which the capercaillie exists naturally (hereinafter 'natural populations'), and from birds constituting two stocks at breeding centres (hereinafter 'farm populations'). Natural populations were sampled in five regions of Poland: the Augustowska Primaeval Forest (53°54'52"N; 23°12'45"E) in the northeastern part of Poland (hereinafter PA); the Solska Primaeval Forest (50°25'0"N; 23°3'45"E) in the Lublin region of eastern Poland (LUB); and in the species’ three Carpathian strongholds, i.e. the Gorce National Park (GOR) (49°36'38"N; 20°3'45"E), the Polish Tatra Mountains National Park (TAT) (49°15'0"N; 19°56'0"E) and Babia Góra National Park (BPN) (49°39'40"N; 19°33'36"E), and beyond Poland: in two regions of Russia (Kirov Oblast (R-K, 58°36'0"N; 49°39'0"E) and the Ukhta region of the Komi Republic (R-U, 63°34'0"N; 53°42'0"E)); and in the southern part of Norrbotten County in Sweden (denoted as SWE), around Arvidsjaur (65°35'0"N; 19°7'0"E) (Fig 1). The numbers of samples collected in each season from each natural population in Poland are as reported in Table A in S1 File. The samples from the Augustowska Primaeval Forest were collected between 2006 and 2010 before the introduction programme there was commenced with in 2012. Birds were also sampled from two breeding centres of the species in Poland: in Wisła Forest District (WIS) (49°38'50"N; 18°52'3"E) and in Leżajsk Forest District (LEZ) (50°15'40"N; 22°25'10"E) (Fig 1).

Sampling in the case of all the natural populations in Poland was entirely non-invasive, involving feathers (*N* = 102) and faeces (*N* = 814). After being collected in the field, feathers were stored in paper envelopes or plastic vials, while after delivery to the laboratory they were kept in a freezer at -4°C. Faeces were collected in 50 ml Falcon Conical Centrifuge Tubes (distribution in Poland by GenoPlast Biochemicals) and covered with silica gel (distribution in Poland by Conbest) to dehydrate the sample. Following delivery to the laboratory and removal of the gel, samples were covered with a new portion of silica gel. Tubes with these contents were then stored in a freezer at -72°C. Samples from R-K were feathers, obtained from 33 birds captured in the wild (Kirov Oblast, Russia) in 2014, and transported to the Głęboki Bród Forest District as part of a conservation programme (Project LIFE11 NAT/PL/428 'The Active Protection of Lowland Populations of Capercaillie in the Bory Dolnośląskie Forest and Augustowska Primaeval Forest'). Moulted contour feathers were collected directly from boxes in which birds had been transported, being placed in tubes with 96% alcohol immediately after collection. Each bird was transported in a separate box. From the Ukhta region we also obtained 12 muscle-tissue samples from hunted birds. These were stored frozen. Feathers were collected from 14 net-captured birds from Sweden, later transported to Ruszów Forest District as part of a conservation programme for the species (Project LIFE11 NAT/PL/428). Feathers were collected directly from the boxes in which birds had been transported, and placed in tubes with 96% alcohol immediately after collection. Samples from birds from Sweden and Russia were collected with permission granted by the General Director of Environmental Protection (Permit number: DZP-WG.6401.03.14.2014.km.2, dated 15.04.2014, and ZP-WG.6401.03.2.2014.km, dated 12.02.2014). We obtained samples of faeces from WIS and LEZ with each bird kept in its own separate pen, providing a faecal sample that was placed in a tube and stored frozen. Only samples from birds constituting breeding stock were collected. We obtained samples from 21 birds from WIS and 24 from LEZ. Overall, a total of 1020 samples for genetic analysis were collected.

### DNA extraction

DNA extractions from feathers were performed using QIAamp DNA Mini Kits (QIAGEN, distribution in Poland by Syngen), or using NucleoSpin Tissue Kits (MACHEREY-NAGEL, distribution in Poland by AQUA LAB) in line with a standard protocol. DNA extraction from muscle tissue samples was also performed with QIAamp DNA Mini Kits (QIAGEN, distribution in Poland by Syngen Biotech).

DNA from faeces was extracted using NucleoSpin Soil Kits (MACHEREY-NAGEL, distribution in Poland by AQUA LAB), using the manufacturers' protocol, except for the fact that double the volume of lysate was used for each sample.

Several measures were taken in association with the DNA isolation process to minimize potential contamination: (i) DNA extraction was performed in a separate room specially designated for the purpose in relation to non-invasive and museum samples, and equipped with UV lamps allowing the workstation, pipettes, tubes, tips and bottles with chemicals to be sterilized. (ii) Prior to extraction, the top of the workstation was cleaned with alcohol. (iii) Each time DNA was extracted from 15 samples and one 'blind' sample (all reagents without biological material) to control for the possibility of contaminated reagents. (iv) We avoided extracting DNA from samples from different populations in the course of a single extraction process. (v) Following the extraction, all pipettes and additional equipment (e.g. scalpels for cutting feathers) were cleaned with alcohol and autoclaved.

### PCR and microsatellite genotyping

All extractions, including the 'blind' samples were subjected to PCR. We aimed to amplify 13 microsatellite loci, i.e. TuT1; TuT2, TuT3, TuT4 (tetranucleotide repeats) and TuD4 and TuD5 (dinucleotide repeats) [29], and 6 tetranucleotide microsatellites isolated from specimens of the black grouse (*Tetrao tetrix*) [30; 31], i.e. TTT1, Bg10, Bg12, Bg14, Bg15, Bg16 and Bg18. Microsatellites were amplified in three multiplex reactions, i.e. MIX A containing primers for amplification of the Bg16, TTT1, TUT2 and Bg12 loci; MIX B: TuD4, TUT1, TUT4, TUT3 and Bg18; and MIX C: Bg15, Bg14, Bg10, TuD5. Each forward primer was labelled with one or other of the fluorescent dyes Dye2, Dye3 and Dye4 (from WellRead Dyes, distribution in Poland by Sigma-Aldrich Poland). The reaction mixture contained 1.5 μl of the mixture of primers ('forward' and 'reverse' for each locus, 2 pmol/μl), 7.5 μl PCR MasterMix (QIAGEN, distribution in Poland by Syngen Biotech), and 1–3 μl of DNA extract, depending on the source of the DNA. Thus, 1 μl of DNA was used from muscle tissue samples and feathers stored in alcohol or collected from boxes, 2 μl of DNA extract from non-invasively collected feathers, and 3 μl of DNA obtained from faeces. In the latter case 0.3 μl of PCR anti-inhibitor (DNA GADAŃSK, distribution in Poland by Blirt) was added. The reaction mix was made up to 15 μl final volume with water for PCR (SIGMA-ALDRICH, distribution in Poland by Sigma-Aldrich Poland). The reactions were performed in the following conditions: 15 min at 95°C, 40 cycles of 30 s at 94°C, 90 s 57°C, 90 sec 72°C 1 cycle: 30 s at 94°C, 90 s 57°C, 10 min at 72°C. As in the case of the extraction process, we sought to control contamination in the course of PCR. Thus, alongside the DNA extracts, each PCR series received a 'blind' sample (with all reagents but no DNA). In addition, as the reaction was prepared for, similar measures as with extraction were taken to minimise the problem of contamination. The genotyping analyses were performed using a CEQ 8000 sequencer (BECKMAN COULTER, distribution in Poland by Comesa-Polska).

Several measures were taken to obtain reliable genotypes: (i) if, during analysis in the sequencer, the PCR product was identified in a 'blind' sample from the extraction process, this set of extracts was excluded from further analysis and the extraction of these samples was repeated; (ii) if, during analysis in the sequencer, the PCR product was identified in a 'blind' sample from the PCR process, this set of PCRs was excluded from further analysis and the PCR for these extracts was repeated; (iii) for each DNA extract all three multiplex PCR reactions were repeated twice. All extracts lacking the PCR product in the two reactions were excluded from further analysis. Similarly, extracts with two identical genotypes in both independent PCRs were classified as successfully genotyped. All the extracts showing signs of contamination (more than two microsatellite alleles at particular loci) were excluded from further analysis. Two additional PCRs were performed in the case of differences between genotypes which could be explained by typical technical problems observed frequently during the microsatellite genotyping of non-invasive samples (i.e. 'allelic drop-out' or 'false alleles' [32]). Consensus genotypes were then created on the basis of the genotypes obtained in all four reactions. The extracts showing evidently different genotypes in successive PCR reactions were excluded from further analysis. For the remaining genotypes, genotyping error rates were estimated using GIMLET 1.3.3 [33], on the basis of the results of fourfold amplification.

### Assigning samples to individuals

In the case of samples from the breeding centres (LEZ, WIS), feathers collected from transporting boxes (R-K, SWE) and samples from hunted individuals (R-U) it was clear that each sample belonged to an individual bird. However, in the case of non-invasive samples collected from the natural population in Poland several samples could have belonged to a single individual. We assumed that the presence of identical microsatellite genotypes in two or more independent samples attested to samples belonging to the same individual. Although we attempted to minimize problems with 'allelic drop-out' and 'false alleles' (see PCR and microsatellite genotyping), we were aware that small differences between genotypes from different samples could still be observed, even if they belonged to a single individual. We assumed that differences at less than three loci, which could be explained by 'allelic drop-out' or a 'false allele', guaranteed that the samples belonged to the same individual. Comparisons of genotypes were performed using GenAlEx v. 6.501 [34; 35], on the basis on nine microsatellite loci (see Results).

### Statistical analysis

To identify possible 'null alleles', 'large allele drop-out' and typographic errors, we analysed microsatellite genotypes in MicroChecker v. 2.2.3 [36]. The percentage of missing data was calculated using GenAlEx v. 6.501 [34; 35]. For each locus within each population, the deviation from the Hardy-Weinberg equilibrium (hereafter *HWE*) and linkage disequilibrium (*LD*) were assessed using Fisher's exact test in Genepop v.4 [37; 38] with the following settings: 10,000 dememorisation, 1000 batches and 10,000 iterations.

For each locus within each population and for a combination of 9 loci, Probability of Identity (the average probability that two unrelated individuals, randomly sampled from a population, will have the same genotype, otherwise *P*_(ID)_) was calculated using GenAlEx v. 6.501 [34; 35]. Additionally, we also calculated Probability of Identity with account taken of genetic similarity among siblings (*P*_(ID-Sibs)_).

We calculated mean values of standard genetic indices: the number of alleles (*A*), allelic richness—the number of alleles corrected for sample size using the rarefaction method with a sample of 12 individuals (*R*, [39]), 'private alleles' (*P*) and 'private allelic richness' (*P*_1_, the number of private alleles corrected for sample size using rarefaction with a sample of 12 individuals), observed (*H*_O_) and expected heterozygosity (*H*_E_, [40]) and inbreeding coefficient (*F*_IS_). These analyses were performed using GenAlEx to calculate *A*, *P*, *H*_O_ and *H*_E_, FSTAT version 2.9.3.2 [41] (*R* and *F*_IS_), and HP-RARE [42] (*P*_1_). Between-population differences in allelic richness (*R*) and observed (*H*_O_) and expected heterozygosity (*H*_E_) were compared using Tukey's HSD test [43] applied to all pairwise comparisons of population means predicted by one-way ANOVAs. This was accomplished using the *TukeyHSD()* command from the *Stats* package in the R environment [44], with a 0.95 family-wise confidence level.

Genetic differentiation among populations was assessed as *F*_ST_ [45], as based on the Infinite Allele Model of mutation, and as *R*_ST_ [46], as based on the Stepwise Mutation Model (SMM [47]). Pairwise *F*_ST_ values and their significance and overall *F*_ST_ with corresponding 95% confidence intervals were calculated in FSTAT. Overall and pairwise *R*_ST_ were calculated in SPAGeDi 1.4 [48], while significance was tested on the basis of 1000 permutations. Aside from *F*_ST_ and *R*_ST_, we also estimated standardized measures of genetic differentiation: *D*_EST_ [49] and *F'*_ST_ [50]. Pairwise and overall *D*_EST_ were calculated using DEMEtics [51]. Significances among obtained values were tested using the bootstrap method (1000 resamplings), as implemented in DEMEtics. The *P*-values were adjusted to account for multiple comparisons, using Bonferroni correction. *F'*_ST_ was calculated by dividing pairwise *F*_ST_ by the maximum value obtained using RecodeData v.0.1 [52].

The Bayesian-clustering method (STRUCTURE version 2.3.4 [53]) was used to examine how well the predefined 'populations' corresponded with genetic groups (*K*). STRUCTURE was run 15 times for each user-defined *K*, with an initial burn-in of 50,000 and 1,000,000 iterations of the total data set. The admixture model of ancestry and the correlated model of allele frequencies were used. Sampling location was not used as prior information. We next examined ΔK statistics that identify the largest change in the estimates of K produced by STRUCTURE, as ΔK may provide a more realistic estimation of *K* than those based on likelihood [54]. STRUCTURE was run independently for two data sets: (i) only natural populations (user define *K* = 1–8); and (ii) natural and farm populations (user define *K* = 1–10). To visualise the STRUCTURE results we used STRUCTURE HARVESTER 0.6.94 [55]. We then applied CLUMPP 1.1.2 [56] to average the multiple runs given by STRUCTURE and correct for label switching. The output from CLUMPP was visualised with DISTRUCT v 1.1 [57] to display the results.

As the identification of genetic structure in STRUCTURE relies on *HWE* optimisation, and as some of the sampling sites in our study were not in *HWE*, we also obtained an additional representation of the genetic structure using principal component analysis (PCA). We used the R package ADEGENET v1.3.4 [58] to carry out standard PCA. The results of the analysis were presented graphically along the first and second axes, in order of the highest Eigen values. As in the case of STRUCTURE, PCA analysis was also performed for two datasets.

Evidence of recent effective reductions in the sizes of populations was investigated using BOTTLENECK 1.2 [59] in respect of the natural and farm populations of the capercaillie. Microsatellite data were tested using the stepwise mutation model (SMM) and a Two-phase Model (TPM [60]) with 95% SMM and variance of 12% [61]. Significance of heterozygote excess was assessed using the Wilcoxon's sign-rank test. Additionally, we tested for recent population bottlenecks using the *M*-ratio method [62], which uses the ratio of the number of alleles to range in allele size (the *M*-ratio) in order to sample microsatellite loci to detect reduction in effective population size (given that allele size range is reduced more slowly than allelic diversity in cases of bottlenecks). The parameter values followed those recommended by the authors [62]. To test the significance of each population’s generated *M*-values (where *M* = *k/r*, and *k* is therefore the number of alleles per locus and *r –* the allelic size range for that locus across all samples), we used Critical_M.exe [63], which generates a critical *M* value (that which 5% of simulations were below) on the basis of the number of individuals sampled and the number of loci, using 10,000 replicates. The observed *M* values below a critical value suggest a bottleneck. Critical values of M were calculated for three different values of θ (defined as 4Neμ, where Ne is the effective population size and μ is the microsatellite mutation rate), i.e. 0.2, 1 and 2, corresponding with long-term equilibrium population sizes of 100, 500 and 1,000, while there was a common estimate of the microsatellite mutation rate = 0.0005 mutations/generation/locus [62]. The percentage of mutations larger than a single step (pg) was set to 0.10, and the mean size of mutations larger than a single step (Δg) to 3.5, in line with recommendations [62]. These analyses were performed for each population and for two groups of populations, suggested by STRUCTURE and PCA analysis: GOR&BPN&TAT and R-K&R-U.

## Results

Among 916 non-invasive samples, the first two PCR reactions did not yield amplification products in 293 samples (32%). Among the remaining 623 samples, two identical genotypes were obtained in 174 cases (28%). In the case of 449 remaining samples, two additional PCRs were performed. Following comparison of genotypes from four subsequent amplifications, 30 samples were excluded from analysis due to clear contamination, while 41 samples were excluded in line with evident differences among the genotypes obtained in subsequent PCR reactions. For the remaining 378 samples, consensus genotypes from four PCR reactions were constructed. Analysis in GIMLET indicated low genotyping error: the maximum error rate per sample calculated for 378 samples genotyped four times for 13 microsatellite loci was: 0.01–0.05 for 241 samples, 0.08–0.12 for 123 samples, and 0.16–0.19 for 14 samples. The mean genotyping errors per locus were 0.01–0.062.

We found 36.7% of missing data in locus TUT1, 26.3% in locus Bg14 and 23.2% in locus Bg15. The presence of missing data was not interlinked with sample type (i.e. non-invasive or invasive) and we decided to exclude these loci from further analysis. Locus Bg10 was also excluded on account of the high frequency of null alleles (18%) as indicated by MicroChecker and significant heterozygote deficiency in seven out of ten investigated populations. We therefore obtained a final set of nine microsatellite loci, successfully amplified in 656 samples (552 non-invasively collected faeces and feathers from natural populations in Poland, feathers found in transporting boxes from 47 individuals, 45 faeces from farms, and 12 tissue samples), without missing data and with no indication of the presence of null alleles. Similarly, for these loci MicroChecker did not suggest problems with stuttering and large-allele drop-out.

On the basis of the genotypes obtained at nine microsatellite loci, we assigned non-invasive samples to individuals. In total, we found that the natural populations in Poland supported 156 unique genotypes (Table 1), which is to say individuals differing from each other by at least four microsatellite loci. In all populations, including the farm population and those from Russia and Sweden we had 260 individuals for investigation (Table 1).

**Table 1:** Summary of the genetic diversity and heterozygosity indices at 9 microsatellite loci among the capercaillie from 8 natural populations and 2 breeding centres (*n* = 260). *N*–number of unique genotypes identified in non-invasive samples (see Table A in S1 File for details) or number of sampled individuals (see Materials and methods); *A*–mean number of alleles per locus; *R*–mean allelic richness; *P*–mean number of private alleles; *P*_*1*_ –mean private allelic richness; *H*_*O*_–heterozygosity observed; *H*_E_–heterozygosity expected; *HWE*–*P*-values for HWE exact test for heterozygote deficiency/excess (ns—non-significant (*P*>0.05)); *F*_IS_–inbreeding coefficient (no *F*_IS_ value proved significant after Bonferroni correction, 1800 randomization, adjusted *P*-value = 0.00056); *N*_C_—estimated census size of natural populations (given as number of individuals).

	*N*	*A*	*R*	*P*	*P*_*1*_	*H*_*O*_	*H*_*E*_	*HWE*	*F*_*IS*_	*N*_C_
PA*	29	5.78	4.89	0.11	0.16	0.661	0.663	ns	0.020	50–70 (2005)^1^; 30–40 (2010)^2^
LUB	20	3.67	3.40	0.00	0.00	0.564	0.537	ns	-0.025	135–150 (2006)^3^; 100–130 (2013)^4^
GOR	44	3.89	3.46	0.00	0.00	0.593	0.583	0.006	-0.007	25–30 (2009)^5^; 30–35 (2014)^6^
BPN	35	4.78	4.06	0.11	0.16	0.578	0.616	ns	0.077	22 (2009)^5^; 35–50 (2014)^6^
TAT	28	5.56	4.89	0.00	0.04	0.671	0.669	ns	0.015	50–70 (2009)^5^; (2014)^6^
R-U	12	4.89	4.89	0.22	0.30	0.722	0.688	0.005	-0.006	4 000 000
R-K	33	6.67	5.49	0.56	0.31	0.704	0.673	0.028	-0.030	
SWE	14	7.00	6.70	0.22	0.34	0.746	0.719	ns	-0.048	200 000
LEZ	24	5.67	4.83	0.33	0.27	0.581	0.628	0.043	0.096	
WIS	21	7.11	6.30	0.22	0.18	0.736	0.750	0.003	0.043	

*PA—Augustowska Primaeval Forest; LUB—Solska Primaeval Forest; GOR—Gorce National Park; TAT—Polish Tatra National Park; BPN—Babia Góra National Park; R-K—Russia, Kirov Oblast; R-U—Russia, Ukhta region in Komi Republic; SWE—Sweden, Norrbotten County; WIS—the breeding centre for capercaillie in Wisła Forest District; LEZ—the breeding centre for capercaillie in Leżajsk Forest District;

^1^[16],

^2^[14],

^3^[17],

^4^[15],

^5^[12],

^6^[13]

For 36 locus x locus combinations there were three cases of significant linkage disequilibrium among the investigated loci after Bonferroni correction (adjusted *P*-value for 5% nominal level was 0.000139, 7200 permutations). Linkage disequilibrium was found in GOR between locus Bg16 and TUD5, and BG16 and TUT2, and between TTT1 and Bg18 in LEZ.

Per locus Probability of Identity (*P*_(ID)_) ranged from 0.03 (locus TUD5 in SWE) to 0.588 (TTT1 in LUB), although in a majority of cases *P*_(ID)_ was lower than 0.2 (Table C in S1 File). For the combination of nine loci, both *P*_(ID)_ and *P*_(ID-Sibs)_ were lower than or equal to 0.001, except in the case of LUB, GOR and BPN where *P*_(ID-Sibs)_ values were 0.004, 0.003 and 0.002, respectively. Hence, the expected number of different individuals with the same genotype was very low.

In three populations (LUB, TAT, SWE) all nine loci were in *HWE* (Table C in S1 File). In the majority of the remaining populations we found from one to two loci deviating from *HWE* as a consequence of both heterozygote deficiency and excess. The largest number of loci deviating from *HWE* (all due to heterozygote deficiency) was found in the WIS farm population. A significant *F*_IS_ value was found at BPN only in the case of locus Bg12 (Table C in S1 File).

The indicators of genetic diversity (Table 1), based on numbers of alleles (mean number of alleles (*A*), mean allelic richness (*R*), mean number of private alleles) provided a basis for the division of the natural populations into three groups. The group of lowest genetic diversity comprises LUB and GOR (*A*<4.0; *R*<3.5; no private alleles). The group of moderate genetic diversity comprises PA, BPN, TAT and R-U (*A* = 4.8–5.8; *R* = 4.0–4.9), albeit with R-U having a clearly higher mean number of private alleles (*P* = 0.22; *P*_1_ = 0.30) than each of the three Polish populations (*P* = 0.00–0.11; *P*_1_ = 0.04–0.16). The group with the highest values for genetic diversity indices (*A*>6.6; *R*>5.4; number of private alleles >0.22) consists of R-K and SWE. We found that mean *R* values were significantly lower (*P*<0.05) in BPN, GOR and LUB than in R-K and SWE.

Levels of heterozygosity corresponded with allelic diversity. The lowest *H*_O_ (<0.60) values were found in LUB, GOR and BPN, while values were moderate (0.66–0.67) in PA and TAT, and highest (>0.70) in R-U, R-K and SWE. In general, the highest genetic diversity among natural populations was found for SWE, the lowest for LUB (Table 1). We did not find significant differences in *H*_O_ and *H*_E_ values between pairs of populations.

Three out of eight natural populations (GOR, R-U, R-K) were not in *HWE* due to heterozygote excess, as indicated by negative *F*_IS_ values. None of the overall *F*_IS_ values proved significant after Bonferroni correction (Table 1). In the case of the farm populations, WIS supported higher genetic diversity than LEZ in terms of both the number of microsatellite alleles and the level of heterozygosity. However, LEZ was found to support a larger number of private alleles than WIS.

We found significant genetic differentiation among the investigated populations (overall *F*_ST_ = 0.159 (95%CI 0.124–0.196); overall *R*_ST_ = 0.137 (*P*<0.05); overall *D*_EST_ = 0.376 (*P*<0.001)). All pairwise *F*_ST_ comparisons were significant with values ranging from 0.02 to 0.28 (Table 2). Analysis of *R*_ST_ indicated significant differentiation in 36 out of 45 pairwise comparisons. Values of *R*_ST_ ranged from -0.01 to 0.30. Similarly, all *D*_EST_ were significant, ranging from 0.05 to 0.61 (Table D in S1 File). Limited genetic differentiation was found to characterize birds from the different Carpathian strongholds (GOR, BPN and TAT), and in comparison between birds from the two sampling sites in Russia (R-U, R-K). Major genetic differentiation was suggested by all pairwise comparisons involving LUB, and pairwise comparisons made between Polish natural populations and R-U and R-K. SWE proved moderately differentiated from other populations with the smallest *F*_ST_,*D*_EST_ and *F*'_ST_ values found for the comparison with TAT, and the smallest *R*_ST_ found for the comparisons with PA and GOR (Table 2 and A in S1 File). Where the farm populations were concerned, LEZ proved more distinct from the natural populations than WIS. In general, standardised measures of differentiation assumed slightly higher values than *F*_ST_ and *R*_ST_, albeit with all measures suggesting a similar pattern of differentiation.

**Table 2:** Genetic differentiation among 8 natural populations and 2 breeding centre populations (*n* = 260) of capercaillie. Above diagonal–*F*_ST_ [45], below diagonal–*R*_ST_ [46]. All *F*_ST_ values are significant after Bonferroni correction (900 randomizations, adjusted *P*-value = 0.0011). Significant values of *R*_ST_ (1000 permutations) are shown in bold. Overall *F*_ST_ = 0.159 (95%CI 0.124–0.196); overall *R*_ST_ = 0.137 (*P*<0.05).

	PA	LUB	GOR	BPN	TAT	R-U	R-K	SWE	LEZ	WIS
PA*		0.19	0.13	0.15	0.11	0.20	0.22	0.10	0.20	0.09
LUB	**0.09**		0.25	0.24	0.21	0.28	0.28	0.20	0.24	0.19
GOR	0.02	**0.11**		0.07	0.05	0.24	0.24	0.12	0.20	0.13
BPN	**0.06**	**0.17**	0.02		0.02	0.22	0.22	0.11	0.18	0.08
TAT	**0.08**	**0.19**	**0.03**	-0.01		0.18	0.19	0.06	0.13	0.07
R-U	**0.11**	**0.24**	**0.13**	**0.21**	**0.23**		0.05	0.13	0.21	0.10
R-K	**0.16**	**0.25**	**0.20**	**0.28**	**0.30**	0.03		0.16	0.22	0.12
SWE	0.03	**0.16**	0.02	**0.08**	**0.09**	**0.09**	**0.16**		0.08	0.08
LEZ	**0.19**	**0.14**	**0.18**	**0.27**	**0.28**	**0.24**	**0.30**	**0.15**		0.12
WIS	0.03	0.03	**0.05**	**0.11**	**0.13**	**0.12**	**0.18**	0.04	**0.06**	

*PA—Augustowska Primaeval Forest; LUB—Solska Primaeval Forest; GOR—Gorce National Park; TAT—Polish Tatra National Park; BPN—Babia Góra National Park; R-K—Russia, Kirov Oblast; R-U—Russia, Ukhta region in Komi Republic; SWE—Sweden, Norrbotten County; WIS—the breeding centre for capercaillie in Wisła Forest District; LEZ—the breeding centre for capercaillie in Leżajsk Forest District

**Fig 2:**
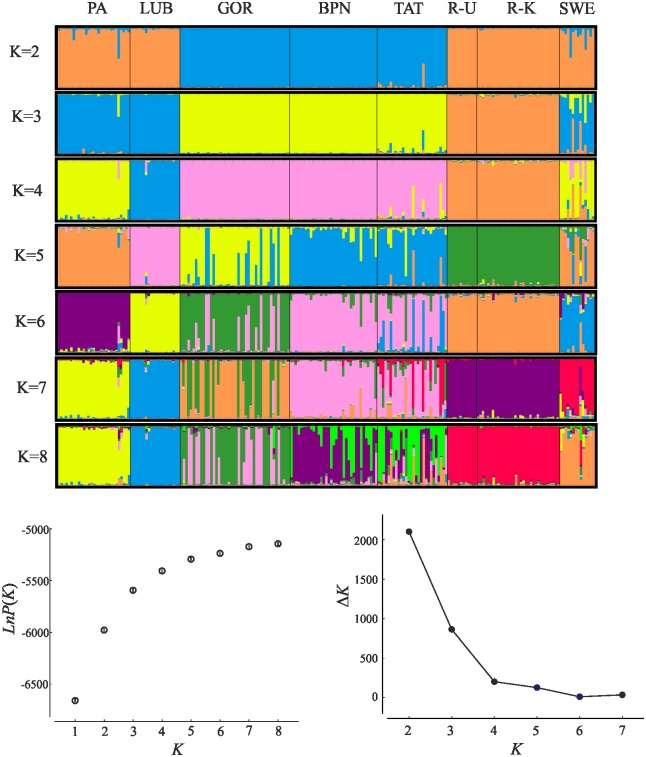
Results of analysis in STRUCTURE for natural populations. Bar plots, estimated mean likelihoods of each number of genetic clusters (bars are SD—only given when exceeding the width of dots) and Δ*K* curves as a function of *K* are presented. In bar plots each individual is represented by a vertical bar partitioned into segments. The length of each segment describes the estimated membership proportions to each of the genetic clusters. Δ*K* suggested a division into two or three genetic clusters. PA—Augustowska Primaeval Forest; LUB—Solska Primaeval Forest; GOR—Gorce National Park; TAT—Polish Tatra National Park; BPN—Babia Góra National Park; R-K—Russia, Kirov Oblast; R-U—Russia, Ukhta region in Komi Republic; SWE—Sweden, Norrbotten County.

Our STRUCTURE analysis for eight natural populations indicated a gradual increase of mean likelihoods from *K* = 1 to *K* = 8 with low variance among iterations (Fig 2). The observed genetic variability was best explained, per Δ*K*, at *K* = 2, but *K* = 3 also had high Δ*K*. Evanno et al. [54] method usually finds the uppermost level of genetic structure within the given dataset and frequently outputs *K* = 2 as the best solution. Hence, we also reported bar plots for higher values of *K*. For *K* = 2 natural populations were divided into 'lowland' (PA, LUB, R-U, R-K, SWE) and 'Carpathian' (GOR, BPN, TAT) groups. Increased *K* further separates R-U and R-K from the 'lowland group' (*K* = 3), LUB from PA (*K* = 4); and dividing individuals from GOR into two genetic clusters—one of them also being present in the two other Carpathian populations (*K* = 5 and *K* = 6). The admixture of individuals from SWE, initially included in the 'lowland group' (*K* = 2), gradually increased, showing connections with PA and the Russian populations (*K* = 4 and 5) and TAT (*K* = 6 and 7).

**Fig 3:**
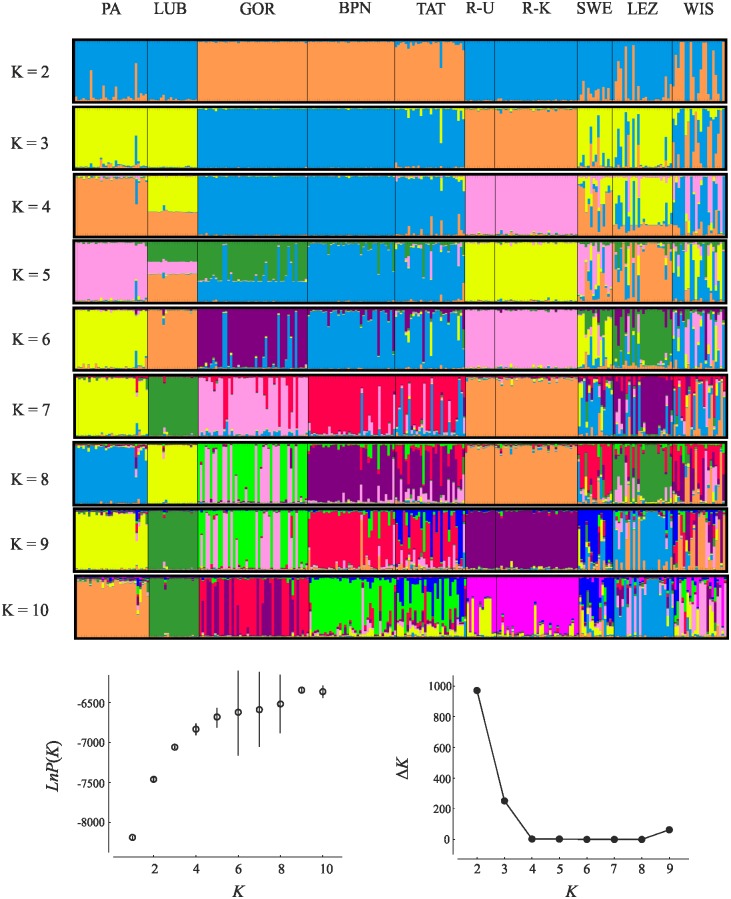
Results of analysis in STRUCTURE for all populations investigated. Bar plots, estimated mean likelihoods of each number of genetic clusters (bars are SD—only given when exceeding the width of dots) and ΔK curves as a function of K are presented. In bar plots each individual is represented by a vertical bar partitioned into segments. The length of each segment describes the estimated membership proportions to each of the genetic clusters. ΔK suggested a division into two or three genetic clusters. PA—Augustowska Primaeval Forest; LUB—Solska Primaeval Forest; GOR—Gorce National Park; TAT—Polish Tatra National Park; BPN—Babia Góra National Park; R-K—Russia, Kirov Oblast; R-U—Russia, Ukhta region in Komi Republic; SWE—Sweden, Norrbotten County; WIS—the breeding centre for capercaillie in Wisła Forest District; LEZ—the breeding centre for capercaillie in Leżajsk Forest District.

Our STRUCTURE analysis for all investigated populations, including the farm populations, revealed a similar pattern of structuring to that of the natural populations (Fig 3). Mean likelihoods increased from *K* = 1 to *K* = 9 with high variance among iterations for *K* = 6–8. Δ*K* indicated the most likely population structure for K = 2 and 3, but an increase at Δ*K* = 9 was also observed. Both breeding centres were found to include individuals from the 'lowland' and 'Carpathian' clusters together with some of admixed origin (*K* = 2). However, for higher *K* (3–6) we identified individuals from the Russian and Carpathian clusters in WIS, whereas birds from LEZ were gradually included in a separate genetic cluster (*K* = 5–10) with some indications of the presence of the gene pool from WIS (Fig 3).

**Fig 4:**
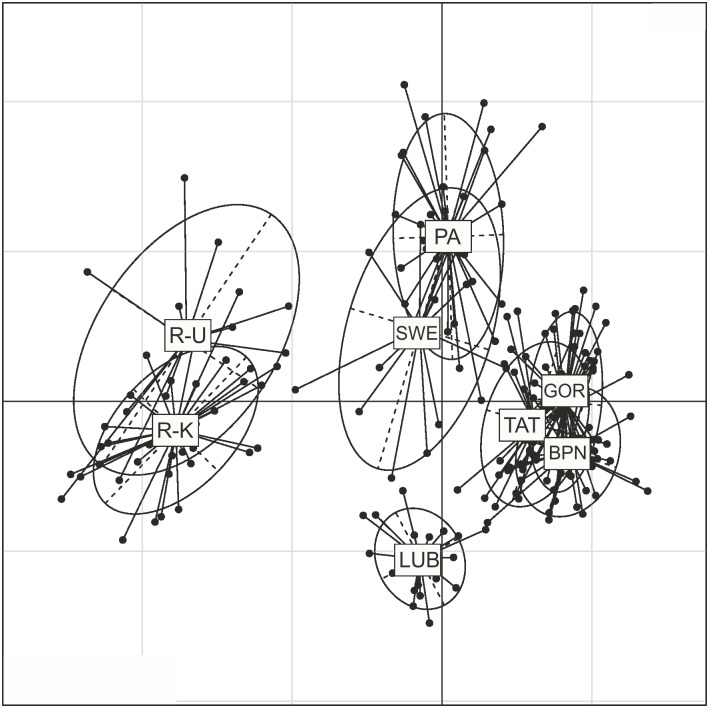
Principal component analysis of the capercaillie genotypes from 8 natural populations. The results are plotted along the first and second axes by reference to the highest Eigen values. PA—Augustowska Primaeval Forest; LUB—Solska Primaeval Forest; GOR—Gorce National Park; TAT—Polish Tatra National Park; BPN—Babia Góra National Park; R-K—Russia, Kirov Oblast; R-U—Russia, Ukhta region in Komi Republic; SWE—Sweden, Norrbotten County.

**Fig 5:**
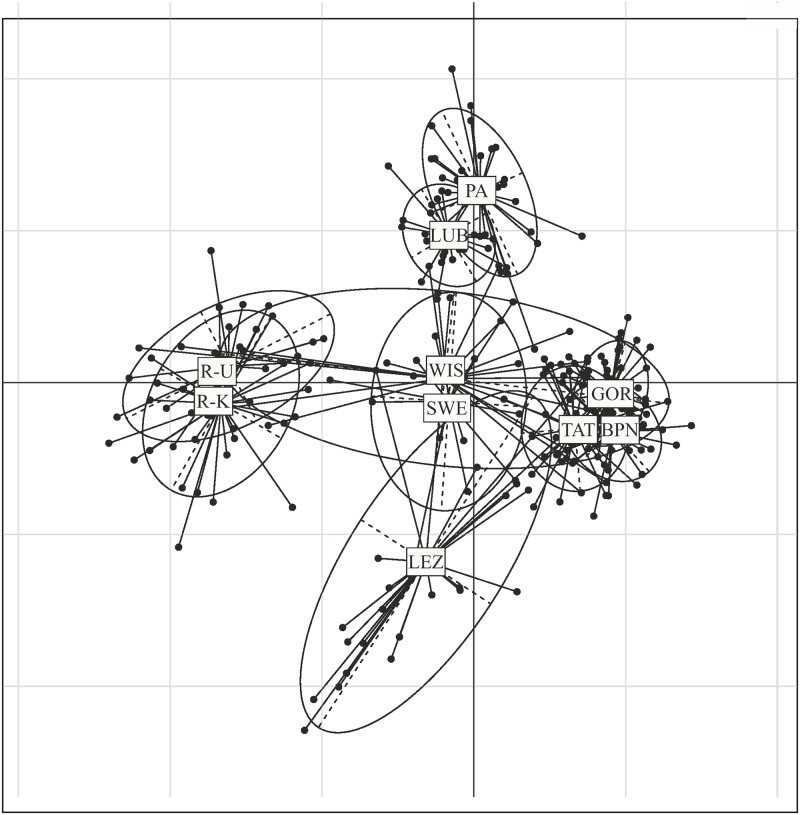
Principal component analysis of the capercaillie genotypes from all investigated populations. The results are plotted along the first and second axes by reference to the highest Eigen values. PA—Augustowska Primaeval Forest; LUB—Solska Primaeval Forest; GOR—Gorce National Park; TAT—Polish Tatra National Park; BPN—Babia Góra National Park; R-K—Russia, Kirov Oblast; R-U—Russia, Ukhta region in Komi Republic; SWE—Sweden, Norrbotten County; WIS—the breeding centre for capercaillie in Wisła Forest District; LEZ—the breeding centre for capercaillie in Leżajsk Forest District.

PCA supported the division of natural populations into four genetic groups (Fig 4), i.e. (i) Russia (R-U and R-K); (ii) Northern (PA and SWE); (iii) Carpathian (GOR, BPN, TAT); and (iv) LUB. The inclusion of farm populations in the analysis led to a regrouping of LUB to PA, while distributing individuals from WIS between the Russian and Carpathian birds (Fig 5). Additionally, birds from LEZ seem to form two genetic groups, i.e. a first one related to the Carpathian population, and a second genetically different from the populations investigated in our study (Fig 5).

All Polish natural populations exhibited patterns consistent with recent bottlenecks (Table 3). The most pronounced effects were found in two of the Carpathian strongholds: GOR and BPN; but also for all Carpathian strongholds included in a single group (GOR&BPN&TAT). We also found signs of a bottleneck in R-U (but not if we combine samples from both Russian sites (R-K&R-U)) and in the breeding stock from LEZ (Table 3). None of the tests suggested a bottleneck effect in R-K and SWE, or breeding stock in WIS.

**Table 3:** Summary of parameters and results for M-ratio analysis and BOTTLENECK 1.2 software used to detect significant reduction in effective population sizes. *N*—sample size; θ = 4Neμ; Ne—pre-bottleneck effective population size, corresponding to given value of θ and mutation rate μ = 10–5; Simulation results—percentage of times when smaller M-ratio at equilibrium is expected, MC—critical value of M (95% of equilibrium values of M should be above MC), Heterozygote excess—P-value of Wilcoxon sign-rank test for heterozygote excess relative to population in mutation-drift equilibrium).

Population	*N*	M-ratio					Bottleneck	
		*N*_e_	θ	average *M*-ratio	*M*_C_	Simulation results (%)	Mutation model	Heterozygote excess
PA*	29	**100**	**0.2**		**0.826**	**1.61**		
		500	1.0	0.787	0.768	7.72	TPM^1^	0.285
		1000	2.0		0.736	17.2	SMM^2^	0.787
LUB	20	**100**	**0.2**		**0.827**	**0.34**		
		**500**	**1.0**	0.740	**0.767**	**2.33**	TPM	0.125
		1000	2.0		0.728	6.99	SMM	0.589
GOR	44	**100**	**0.2**		**0.828**	**0.00**		
		**500**	**1.0**	0.649	**0.772**	**0.04**	**TPM**	**0.002**
		**1000**	**2.0**		**0.743**	**0.19**	SMM	0.285
BPN	35	**100**	**0.2**		**0.827**	**0.05**		
		**500**	**1.0**	0.671	**0.772**	**0.02**	TPM	0.101
		**1000**	**2.0**		**0.735**	**0.70**	SMM	0.203
TAT	28	**100**	**0.2**		**0.828**	**2.04**		
		500	1.0	0.795	0.770	9.50	TPM	0.285
		1000	2.0		0.735	21.16	SMM	0.849
GOR&BPN&TAT	107	**100**	**0.2**		**0.829**	**0.69%**		
		**500**	**1.0**	0.766	**0.776**	**3.93%**	TPM	0.410
		1000	2.0		0.749	8.02%	SMM	0.986
R-U	12	**100**	**0.2**		**0.824**	**2.18**		
		500	1.0	0.792	0.761	10.56	**TPM**	**0.007**
		1000	2.0		0.717	26.05	SMM	0.248
R-K	33	100	0.2		0.830	10.17		
		500	1.0	0.856	0.768	31.03	TPM	0.326
		1000	2.0		0.736	52.06	SMM	0.986
R-U&R-K	45	100	0.2		0.827	14.91%		
		500	1.0	0.872	0.776	38.65%	TPM	0.231
		1000	2.0		0.738	61.80%	SMM	0.986
SWE	14	100	0.2		0.824	20.02		
		500	1.0	0.887	0.746	50.30	TPM	0.632
		1000	2.0		0.720	75.20	SMM	0.875
LEZ	24	**100**	**0.2**		**0.822**	**0.74**		
		**500**	**1.0**	0.765	**0.767**	**4.79**	TPM	0.589
		1000	2.0		0.729	11.65	SMM	0.990
WIS	21	100	0.2		0.829	16.15		
		500	1.0	0.876	0.768	44.34	TPM	0.125
		1000	2.0		0.728	66.83	SMM	0.981

^1^TPM- two-phase model;

^2^SMM—stepwise model;

*PA—Augustowska Primaeval Forest; LUB—Solska Primaeval Forest; GOR—Gorce National Park; TAT—Polish Tatra National Park; BPN—Babia Góra National Park; R-K—Russia, Kirov Oblast; R-U—Russia, Ukhta region in Komi Republic; SWE—Sweden, Norrbotten County; WIS—the breeding centre for capercaillie in Wisła Forest District; LEZ—the breeding centre for capercaillie in Leżajsk Forest District.

## Discussion

Identification of Conservation Units can assist conservationists and wildlife managers involved in the conservation of endangered species to focus management efforts on specifically defined areas and populations [26; 64]. In operational terms, Evolutionarily Significant Units should be identified as reciprocally monophyletic units for mtDNA haplotypes, being simultaneously significantly divergent for nuclear allele frequencies. Management Units are usually identified on the basis of a significant *F*_ST_ at nuclear loci, for example microsatellite alleles [64]. We focused our research on estimation of genetic differentiation in nuclear microsatellites. We detected a clear division of the natural populations into genetic groups described as 'lowland' (PA—the Augustowska Primaeval Forest, LUB—the Solska Primaeval Forest, Russia and Sweden) and 'mountain' (the Polish Carpathians). Analysis of genetic differentiation and genetic structure thus gave rise to the suggestion that the Polish lowland populations (of the Augustowska and Solska Primaeval Forests) and the population from the Polish Carpathians should be treated at least as separate Management Units. This result corroborates an observation [65] which indicated that the Western Carpathian population is genetically differentiated from the boreal capercaillie. A similar pattern of genetic difference is shared by its sister species, the black grouse [66] and hazel grouse (*Terastes bonasia*) [67; 68]. These phenomena among grouse species can be explained by the existence of a Carpathian glacial refugium during the Last Glacial Maximum, ca. 27 500–19 000 YBP [69]. Alternatively, analysis of the mitochondrial genome indicates that the Western Carpathian capercaillie belong to the 'boreal' lineage, distributed around major areas of Europe [22–24]. It is possible that the 'boreal' lineage consists of subgroups of capercaillies that are 'lowland' (from Poland, Sweden and Russia) or else Western Carpathian.

Our STRUCTURE and PCA analysis revealed deeper divisions of the investigated natural populations. First, when *K* = 3, the Augustowska and Solska Primaeval Forests (PA and LUB) were grouped together with the population from Sweden, and this group was separated from Russian populations. The existence of genetic differentiation between Russian and Swedish birds was also supported by PCA. This corresponds with a previous study [70], indicating that birds from southern Norway are less differentiated genetically from Central European populations than from the capercaillie from Russia. Alternatively, recent microsatellite analysis has grouped birds from Sweden and Russia into a single cluster [65]. It is possible that this discrepancy could be explained by the postglacial recolonisation of the Scandinavian Peninsula from two directions: through a land bridge in the southwest and through Finland from the northeast [71; 72]. As a result, in some species, including other woodland grouse (i.e. hazel grouse [73]), two genetic populations are often identified. It is then possible that our study sampled a different genetic group from what was investigated previously [65]. Second, when *K*≥3, isolation of the Solska Primaeval Forest (LUB) was evident, an observation also supported by PCA. Clearly, isolation and subsequent genetic drift have led to the genetic differentiation of these birds from other lowland populations—a conclusion supported by high values for *F*_ST_, *R*_ST_ and *D*_EST_. Hence, we suggest that the Solska Primaeval Forest and its population should be assigned to a separate MU.

Regarding genetic diversity, our result supports a previous study [25], which demonstrated that the capercaillie from Poland have lower genetic diversity than do larger, continuous populations in Russia and Sweden. The fragmentation of forests and habitat deterioration divide populations of these sedentary birds into smaller sub-populations of limited size, enhancing the effects of genetic drift. Similar effects have also been found in other European grouse species, i.e. the black grouse [64, 74; 75] and hazel grouse [67; 68]. All natural populations from Poland provide evidence of bottlenecks having been experienced. Although the result of this analysis should be treated with caution, as we analysed only nine microsatellite loci, the decrease in the number of individuals in Poland in the 20th century is well documented [10]. Hence, our results confirm how known demographic trends may have impacted genetic diversity.

Surprisingly, genetic diversity was lowest in Poland’s largest population, i.e. that of the Solska Primaeval Forest (LUB) (with 100–130 birds as of 2013 [15]), inhabiting an extensive (ca. 1300 km^2^) area. A low level of genetic diversity for this population was suggested previously [25] as a result of this forest’s long-term (several-century) isolation [10]. Our observation supports the idea that isolation and subsequent genetic drift are potent factors shaping levels of genetic diversity in the capercaillie [70, 76]. Alternatively, the size of the Solska Primaeval Forest population may have been overestimated due to limitations in the use of lek counts to estimate population size [77].

A low level of genetic diversity also characterised birds from the Gorce and Babia Góra National Parks (GOR and BPN). These are considered to join the Polish Tatra National Park (TAT) within the Western Carpathian population [13]. Indeed, our comprehensive set of analyse supports the suggestion that Carpathian sites are or have been, at least recently, well connected through gene flow. The mountain forests in Poland are much less fragmented than those in the country’s lowlands. Gene flow probably occurs along forested slopes and lower mountain ridges. Limited genetic differentiation between mountain populations in Poland has also been confirmed in the hazel grouse [67; 68] and alpine population of the black grouse [74]. Our results thus support the suggestion that strongholds of the capercaillie in the Polish Carpathians should be treated as a single Management Unit. We nevertheless found strong evidence for recent bottlenecks affecting the population from the Polish Carpathians. The Gorce Mountains are separated from other Carpathian populations by an extensive area of unsuitable habitat (i.e., a non-forested area and the large urbanised area of Nowy Targ). We suggest that this separation has led to genetic isolation, resulting in intensive genetic drift. The Gorce National Park (GOR) is the only Polish population with confirmed heterozygote excess. However, this measure of bottleneck indicates that a reduction in numbers has occurred recently. In fact, it is estimated that the number of birds in the Gorce National Park was <20 individuals in the 1980s [10; 78]. Although the population in the Babia Góra National Park (BPN) also showed a low level of genetic diversity, especially in terms of heterozygosity, this stronghold could still be connected with areas supporting the capercaillie population across the border in Slovakia. Indeed, limited genetic differentiation of capercaillie from Slovakia and Poland has been inferred recently [65] The private alleles found in the Babia Góra National Park population could indicate possible gene flow from Slovakia, or the capercaillie strongholds in the Polish Carpathians located to the west of BPN (in the Beskid Żywiecki hills).

The highest genetic diversity in natural populations from Poland characterises the Augustowska Primaeval Forest (PA) and the Polish Tatra National Park (TAT). As already mentioned, TAT could be connected with the capercaille population from Slovakia, and observations suggest that current numbers are higher there than in the other Carpathians strongholds. The Augustowska Primaeval Forest is located in northeastern Poland in close proximity to other large forests like the Knyszyńska and Białowieska Primaeval Forests. These forests retain some connections because of the presence of belts of woodland. In the first half of the 20th century there were still capercaillie present, not only in the Augustowska Primaeval Forest, but also in other forests of northeastern Poland [11; 78]. It was only at the end of the 20th century that this species became extinct in the Knyszyńska and Białowieska Primaeval Forests, though the population from the Augustowska Primaeval Forest was then relatively stable demographically, even showing some signs of an increase [14]. At the beginning of the 21st century the Augustowska Primaeval Forest maintained the most genetically diverse Polish population of capercaillie [25]. However, it is estimated that the last 15 years have brought declines in the number of birds at an average annual rate of 3% [14]. Currently, the population is estimated at 30–40 birds. Notwithstanding this small size, the Augustowska Primaeval Forest retains a high level of genetic diversity relative to other natural populations in Poland. This discrepancy may reflect the recent decrease in population size and too little time having elapsed for genetic variability to be affected. High genetic diversity in the forests of northeastern Poland has also been indicated for the hazel grouse [67; 68], and this phenomenon, together with the existence of a genetic substructure, has been advanced as evidence of different colonisation routes of the northeastern forests in the postglacial period. It is also possible that the Augustowska Primaeval Forest sustains gene flow with the populations of capercaillie present in Belarus and Lithuania.

Four Polish breeding centres for the capercaillie have been established in recent years. Three run by the State Forests in its Wisła, Leżajsk and Głęboki Bród Forest Districts [15; 79; 80] and one (breeding grouse in general) at the Wildlife Park in Kadzidłowo [81]. We investigated the breeding stocks at the two centres: in Wisła (WIS) and Leżajsk (LEZ) Forest Districts. The Wisła centre was established in 2002 with breeding stock deriving from both the Polish Carpathians (Gorce Mountains, Żywiecki Beskids and Polish Tatras) and Belarus [80]. Two breeding lines are maintained, with offspring mainly reinforcing the natural population in the Beskids. The Leżajsk centre began in 1993 [79] and presently, its breeding stock derives from Belarus and the Wisła breeding centre (Z. Szkamruk, pers. com.). The analysis of microsatellite markers indicates high genetic diversity, especially in Wisła. Our results indicate the presence of two genetic clusters in WIS, one corresponding genetically with the population from the Polish Carpathians, the other with the 'lowland' cluster. The presence of the 'lowland' cluster in the breeding stock from the Wisła centre reflects genetic similarity between birds from Belarus and Russia. Clearly, the high genetic diversity in this breeding stock results from the presence of two different genetic pools. Despite the breeding isolation of these two lines in the breeding programme pursued at the Wisła centre, we detected the presence of a few individuals of mixed origin. Therefore we suggest there may be inaccuracies between the breeding documentation and pedigree genetic data or wrong assignment of founder individuals to genetic lineages [82]. We speculate that we can no longer assume isolation between breeding lines. Regardless, we recommend separate maintenance of the Belarusian and Carpathian lines, as we found substantial genetic differentiation between Carpathian and lowland capercaillie.

The breeding stock from the Leżajsk centre (LEZ) is also diverse genetically, though the number of microsatellite alleles and heterozygosity were clearly lower than in birds at the Wisła centre. Similarly, we detected the presence of Carpathian and lowland genetic clusters in this breeding population. Unfortunately, there is little information available about the exact origin of the founders. Until recently appropriate breeding documentation was not standard protocol. However, the large number of private alleles suggest that the majority of individuals from the Leżajsk centre originated from some other population(s) not investigated in our study.

## Conclusions

All Polish populations of the capercaillie show reduced genetic diversity and signs of genetic bottlenecks. To preserve the highest level of genetic diversity, all of Poland’s natural populations of the capercaillie should be made subject to conservation measures, as the Polish population of the species constitutes a very diverse gene pool. We found significant genetic differentiation at microsatellite markers between lowland and Carpathian populations. Hence, our genetic data indicate that lowland populations of the capercaillie in Poland (from the Augustowska and Solska Primaeval Forests) and the population from the Polish Carpathians should be assigned to separate Conservation Units (Management Units). Additionally, the high level of genetic differentiation of birds from the Solska Primaeval Forest suggests that this area should also be treated as an independent Management Unit.

## Supporting information

S1 File **Table A. Non-invasive sampling information.** Collected—number of collected samples; Genotyped—number of genotyped samples, GR—successful genotyping ratio for 9 microsatellite loci (see: Results). **Table B. Two-digit genotypes of individuals at nine microsatellite loci. Table C. Microsatellite polymorphisms in the investigated populations**. *N*—number of unique genotypes, identified in non-invasive samples (see Table A for details) or number of sampled individuals (see Material and methods); *A*–mean number of alleles per locus; *H*_*O*_–heterozygosity observed; *H*_E_–heterozygosity expected; *HWE*–*P*-values for HWE exact test for heterozygote deficiency/excess (*—*P*<0.05; ns—non-significant (*P*>0.05))); *F*_IS_–fixation index (*–significant after Bonferroni correction); *P*_(ID)_ by locus—Probability of Identity for each locus; All loci loci *P*_(ID)_/*P*_(ID-Sibs)_–Probability of Identity for combination of 9 loci/ Probability of Identity for combination of 9 loci, taking into account the genetic similarity among siblings. **Table D. Genetic differentiation among 8 natural populations and 2 breeding flocks (*n* = 260) of the capercaillie, estimated using standardized measures: *D***_**EST**_ **and *F*'**_**ST**_.
(DOCX)
