# Supplementary material for: Conservation genetics of the capercaillie in Poland - Delineation of conservation units
Source: PLoS One. 2017 Apr 4;12(4):e0174901. doi: 10.1371/journal.pone.0174901 (PMC5380343; doi:10.1371/journal.pone.0174901)
Supplement: S1 File — Table A. Non-invasive sampling information. Collected—number of collected samples; Genotyped—number of genotyped samples, GR—successful genotyping ratio for 9 microsatellite loci (see: Results). Table B. Two-digit genotypes of individuals at nine microsatellite loci. Table C. Microsatellite polymorphisms in the investigated populations. N—number of unique genotypes, identified in non-invasive samples (see Table A for details) or number of sampled individuals (see Material and methods); A–mean number of alleles per locus; HO–heterozygosity observed; HE–heterozygosity expected; HWE–P-values for HWE exact test for heterozygote deficiency/excess (*—P<0.05; ns—non-significant (P>0.05))); FIS–fixation index (*–significant after Bonferroni correction); P(ID) by locus—Probability of Identity for each locus; All loci loci P(ID)/P(ID-Sibs)–Probability of Identity for combination of 9 loci/ Probability of Identity for combination of 9 loci, taking into account the genetic similarity among siblings. Table D. Genetic differentiation among 8 natural populations and 2 breeding flocks (n = 260) of the capercaillie, estimated using standardized measures: DEST and F'ST. (DOCX) [file pone.0174901.s001.docx]

Supplementary Materials

Table A. Non-invasive sampling information: Collected – number of collected samples; Genotyped – number of genotyped samples, GR ― successful genotyping ratio for 9 microsatellite loci (see: Results)

| Year | 2005 | 2006 | 2007 | 2008 | 2009 | 2010 |  | 2012 | 2013 | 2014 | Total |
| --- | --- | --- | --- | --- | --- | --- | --- | --- | --- | --- | --- |
|  | PA | | | | | | | | | | |
| Collected |  | 41 | 22 | 3 | 5 | 27 |  |  |  |  | 98 |
| Genotyped |  | 22 | 14 | 2 | 2 | 17 |  |  |  |  | 57 |
| GR (%) |  |  |  |  |  |  |  |  |  |  | 58.16 |
|  | LUB | | | | | | | | | | |
| Collected | 15 |  |  | 8 | 60 |  |  |  |  |  | 83 |
| Genotyped | 4 |  |  | 7 | 37 |  |  |  |  |  | 48 |
| GR (%) |  |  |  |  |  |  |  |  |  |  | 57.83% |
|  | GOR | | | | | | | | | | |
| Collected |  |  |  |  | 7 | 58 |  | 126 | 118 |  | 310 |
| Genotyped |  |  |  |  | 3 | 51 |  | 100 | 49 |  | 203 |
| GR (%) |  |  |  |  |  |  |  |  |  |  | 65.48% |
|  | BPN | | | | | | | | | | |
| Collected |  |  |  |  |  | 17 |  | 85 | 102 |  | 204 |
| Genotyped |  |  |  |  |  | 9 |  | 40 | 78 |  | 127 |
| GR (%) |  |  |  |  |  |  |  |  |  |  | 62.25% |
|  | TAT | | | | | | | | | | |
| Collected |  |  |  |  |  |  |  | 89 | 53 | 79 | 221 |
| Genotyped |  |  |  |  |  |  |  | 57 | 24 | 36 | 117 |
| GR (%) |  |  |  |  |  |  |  |  |  |  | 52.94% |
|  | All Polish natural populations | | | | | | | | | | |
| Collected | 15 | 41 | 22 | 11 | 72 | 102 |  | 300 | 273 | 79 | 916 |
| Genotyped | 4 | 22 | 14 | 9 | 42 | 78 |  | 197 | 151 | 36 | 552 |
| GR (%) |  |  |  |  |  |  |  |  |  |  | 60.26% |

Table B. Two-digit genotypes of individuals at nine microsatellite loci.

| **Bg16** | **TTT1** | **TUT2** | **Bg12** | **TUD4** | **TUT4** | **TUT3** | **Bg18** | **TUD5** |
| --- | --- | --- | --- | --- | --- | --- | --- | --- |
| PA |  |  |  |  |  |  |  |  |
| 0707 | 0206 | 0707 | 0505 | 0210 | 0405 | 0607 | 0303 | 0910 |
| 0511 | 0202 | 0304 | 0404 | 0816 | 0205 | 0505 | 0307 | 0610 |
| 0711 | 0202 | 0306 | 0405 | 0210 | 0507 | 0506 | 0303 | 0710 |
| 0507 | 0202 | 0507 | 0404 | 0102 | 0405 | 0505 | 0203 | 0710 |
| 0507 | 0206 | 0303 | 0405 | 0202 | 0204 | 0606 | 0303 | 0407 |
| 1111 | 0202 | 0707 | 0505 | 0101 | 0406 | 0607 | 0202 | 0707 |
| 0707 | 0606 | 0707 | 0405 | 0303 | 0505 | 0606 | 0303 | 0413 |
| 0511 | 0202 | 0507 | 0405 | 0308 | 0205 | 0608 | 0303 | 0409 |
| 0507 | 0202 | 0307 | 0404 | 0208 | 0405 | 0506 | 0203 | 0711 |
| 0711 | 0202 | 0303 | 0405 | 0210 | 0205 | 0505 | 0303 | 0611 |
| 0507 | 0202 | 0407 | 0406 | 0116 | 0304 | 0406 | 0305 | 0406 |
| 0707 | 0202 | 0405 | 0404 | 0108 | 0305 | 0606 | 0303 | 0404 |
| 0508 | 0202 | 0307 | 0405 | 0101 | 0303 | 0506 | 0305 | 0407 |
| 0711 | 0101 | 0708 | 0606 | 0116 | 0405 | 0506 | 0303 | 0710 |
| 0512 | 0202 | 0304 | 0405 | 0101 | 0305 | 0506 | 0305 | 0407 |
| 0711 | 0206 | 0304 | 0000 | 0310 | 0505 | 0506 | 0303 | 0610 |
| 0707 | 0204 | 0305 | 0404 | 0116 | 0303 | 0608 | 0303 | 0404 |
| 0511 | 0102 | 0407 | 0506 | 1616 | 0405 | 0606 | 0304 | 1013 |
| 0811 | 0204 | 0407 | 0404 | 1616 | 0305 | 0406 | 0303 | 0410 |
| 0509 | 0202 | 0405 | 0404 | 0810 | 0304 | 0607 | 0303 | 0610 |
| 0711 | 0202 | 0304 | 0406 | 0108 | 0304 | 0508 | 0203 | 0610 |
| 0707 | 0204 | 0303 | 0505 | 0202 | 0404 | 0506 | 0303 | 0606 |
| 0711 | 0204 | 0305 | 0405 | 0102 | 0405 | 0505 | 0303 | 0610 |
| 0507 | 0202 | 0607 | 0606 | 0316 | 0304 | 0506 | 0305 | 0607 |
| 0708 | 0204 | 0405 | 0104 | 0101 | 0306 | 0708 | 0303 | 0406 |
| 0708 | 0103 | 0304 | 0405 | 0610 | 0405 | 0506 | 0103 | 0606 |
| 0711 | 0206 | 0305 | 0104 | 0108 | 0505 | 0606 | 0203 | 0710 |
| 0707 | 0206 | 0305 | 0105 | 0108 | 0305 | 0508 | 0103 | 0407 |
| 0508 | 0202 | 0307 | 0104 | 0101 | 0305 | 0506 | 0203 | 0410 |
| LUB |  |  |  |  |  |  |  |  |
| 0409 | 0202 | 0304 | 0407 | 0103 | 0404 | 0508 | 0206 | 0711 |
| 0808 | 0202 | 0404 | 0104 | 0105 | 0405 | 0505 | 0206 | 0710 |
| 0809 | 0202 | 0306 | 0104 | 0511 | 0405 | 0505 | 0207 | 1011 |
| 0809 | 0202 | 0304 | 0104 | 0505 | 0405 | 0505 | 0607 | 0710 |
| 0408 | 0202 | 0304 | 0000 | 0105 | 0505 | 0505 | 0205 | 1011 |
| 0808 | 0202 | 0306 | 0404 | 0101 | 0405 | 0505 | 0205 | 0711 |
| 0708 | 0203 | 0305 | 0101 | 0103 | 0405 | 0505 | 0205 | 0709 |
| 0511 | 0303 | 0303 | 0107 | 0101 | 0405 | 0508 | 0202 | 0707 |
| 0408 | 0202 | 0304 | 0404 | 0101 | 0405 | 0508 | 0202 | 1011 |
| 0408 | 0202 | 0303 | 0104 | 1111 | 0405 | 0505 | 0205 | 0710 |
| 0408 | 0202 | 0303 | 0107 | 0305 | 0505 | 0505 | 0202 | 0710 |
| 0808 | 0203 | 0304 | 0104 | 0103 | 0405 | 0508 | 0202 | 0710 |
| 0409 | 0202 | 0304 | 0404 | 1111 | 0404 | 0505 | 0205 | 1111 |
| 0408 | 0203 | 0304 | 0101 | 0103 | 0404 | 0505 | 0202 | 0707 |
| 0508 | 0203 | 0306 | 0101 | 0105 | 0405 | 0505 | 0205 | 0707 |
| 0808 | 0202 | 0406 | 0707 | 0105 | 0505 | 0505 | 0202 | 0710 |
| 0808 | 0202 | 0304 | 0707 | 0103 | 0505 | 0507 | 0202 | 0711 |
| 0409 | 0202 | 0303 | 0104 | 0103 | 0406 | 0507 | 0506 | 0710 |
| 0404 | 0202 | 0303 | 0104 | 0311 | 0505 | 0505 | 0202 | 1010 |
| 0809 | 0202 | 0306 | 0101 | 0105 | 0405 | 0505 | 0507 | 1011 |
| GOR |  |  |  |  |  |  |  |  |
| 0707 | 0202 | 0303 | 0404 | 0101 | 0506 | 0606 | 0304 | 0809 |
| 0707 | 0206 | 0307 | 0404 | 0115 | 0404 | 0606 | 0404 | 0606 |
| 0307 | 0202 | 0507 | 0404 | 0101 | 0306 | 0609 | 0304 | 0809 |
| 0708 | 0104 | 0305 | 0404 | 0101 | 0505 | 0506 | 0304 | 0606 |
| 0707 | 0202 | 0305 | 0404 | 0115 | 0406 | 0909 | 0304 | 0606 |
| 0708 | 0202 | 0303 | 0707 | 0101 | 0306 | 0609 | 0404 | 0608 |
| 0708 | 0204 | 0305 | 0104 | 0115 | 0506 | 0506 | 0304 | 0609 |
| 0708 | 0204 | 0303 | 0404 | 0111 | 0506 | 0506 | 0405 | 0606 |
| 0308 | 0206 | 0305 | 0707 | 0101 | 0506 | 0606 | 0304 | 0608 |
| 0808 | 0202 | 0305 | 0407 | 0115 | 0305 | 0609 | 0405 | 0608 |
| 0708 | 0102 | 0305 | 0101 | 1115 | 0405 | 0506 | 0303 | 0606 |
| 0708 | 0204 | 0305 | 0101 | 1111 | 0406 | 0506 | 0304 | 0506 |
| 0307 | 0206 | 0307 | 0404 | 0115 | 0306 | 0606 | 0304 | 0609 |
| 0707 | 0102 | 0505 | 0104 | 0115 | 0405 | 0509 | 0303 | 0606 |
| 0307 | 0206 | 0305 | 0407 | 0101 | 0505 | 0606 | 0303 | 0608 |
| 0707 | 0202 | 0505 | 0407 | 0115 | 0505 | 0609 | 0304 | 0606 |
| 0808 | 0202 | 0305 | 0407 | 0115 | 0505 | 0406 | 0305 | 0608 |
| 0308 | 0206 | 0305 | 0407 | 0115 | 0505 | 0606 | 0304 | 0608 |
| 0808 | 0202 | 0305 | 0407 | 0115 | 0506 | 0606 | 0304 | 0608 |
| 0707 | 0202 | 0307 | 0407 | 0103 | 0606 | 0609 | 0404 | 0609 |
| 0708 | 0202 | 0103 | 0707 | 0101 | 0506 | 0609 | 0405 | 0609 |
| 0308 | 0206 | 0307 | 0707 | 0115 | 0506 | 0609 | 0305 | 0608 |
| 0307 | 0206 | 0507 | 0404 | 0101 | 0506 | 0606 | 0304 | 0809 |
| 0707 | 0204 | 0305 | 0104 | 1515 | 0405 | 0506 | 0304 | 0606 |
| 0307 | 0206 | 0103 | 0407 | 0101 | 0506 | 0606 | 0304 | 0609 |
| 0708 | 0202 | 0305 | 0407 | 0115 | 0406 | 0609 | 0405 | 0606 |
| 0808 | 0202 | 0505 | 0104 | 0115 | 0508 | 0506 | 0405 | 0606 |
| 0707 | 0104 | 0305 | 0104 | 1115 | 0406 | 0509 | 0304 | 0606 |
| 0708 | 0202 | 0507 | 0404 | 0101 | 0406 | 0509 | 0404 | 0606 |
| 0708 | 0202 | 0507 | 0407 | 0115 | 0506 | 0606 | 0405 | 0808 |
| 0708 | 0204 | 0505 | 0104 | 1515 | 0405 | 0509 | 0405 | 0606 |
| 0808 | 0202 | 0505 | 0404 | 0115 | 0405 | 0509 | 0405 | 0606 |
| 0707 | 0102 | 0305 | 0101 | 1515 | 0404 | 0506 | 0303 | 0606 |
| 0808 | 0202 | 0305 | 0407 | 0115 | 0506 | 0609 | 0405 | 0808 |
| 0707 | 0206 | 0103 | 0707 | 0115 | 0606 | 0606 | 0304 | 0608 |
| 0808 | 0202 | 0303 | 0707 | 0101 | 0606 | 0606 | 0303 | 0608 |
| 0707 | 0101 | 0505 | 0101 | 0115 | 0508 | 0609 | 0303 | 0606 |
| 0708 | 0202 | 0307 | 0407 | 1115 | 0507 | 0506 | 0404 | 0606 |
| 0808 | 0202 | 0305 | 0404 | 0101 | 0606 | 0606 | 0405 | 0808 |
| 0708 | 0102 | 0505 | 0101 | 0115 | 0408 | 0506 | 0304 | 0506 |
| 0808 | 0202 | 0305 | 0404 | 0115 | 0506 | 0606 | 0405 | 0608 |
| 0308 | 0206 | 0305 | 0404 | 0115 | 0506 | 0506 | 0303 | 0608 |
| 0308 | 0606 | 0305 | 0404 | 0101 | 0505 | 0606 | 0303 | 0608 |
| 0707 | 0202 | 0103 | 0404 | 0101 | 0306 | 0406 | 0304 | 0606 |
| BPN |  |  |  |  |  |  |  |  |
| 0308 | 0202 | 0305 | 0104 | 0111 | 0303 | 0608 | 0304 | 0609 |
| 0708 | 0202 | 0305 | 0207 | 0115 | 0308 | 0609 | 0308 | 0606 |
| 0708 | 0102 | 0303 | 0202 | 0101 | 0508 | 0607 | 0303 | 0509 |
| 0708 | 0202 | 0303 | 0101 | 1515 | 0408 | 0606 | 0303 | 0809 |
| 0808 | 0202 | 0307 | 0102 | 0115 | 0306 | 0608 | 0304 | 0606 |
| 0708 | 0202 | 0303 | 0107 | 0115 | 0308 | 0506 | 0303 | 0813 |
| 0808 | 0202 | 0303 | 0707 | 0104 | 0508 | 0606 | 0303 | 0809 |
| 0808 | 0102 | 0303 | 0707 | 0115 | 0608 | 0606 | 0308 | 0608 |
| 0708 | 0202 | 0303 | 0107 | 1515 | 0306 | 0506 | 0404 | 0608 |
| 0808 | 0202 | 0303 | 0707 | 0115 | 0303 | 0508 | 0304 | 0606 |
| 0708 | 0202 | 0303 | 0707 | 0104 | 0303 | 0506 | 0308 | 0608 |
| 0708 | 0202 | 0707 | 0101 | 0411 | 0308 | 0606 | 0404 | 0606 |
| 0708 | 0102 | 0506 | 0707 | 0115 | 0506 | 0608 | 0304 | 0809 |
| 0708 | 0202 | 0307 | 0101 | 0101 | 0306 | 0608 | 0308 | 0608 |
| 0707 | 0102 | 0305 | 0202 | 0115 | 0608 | 0609 | 0305 | 0609 |
| 0708 | 0204 | 0307 | 0202 | 0115 | 0305 | 0606 | 0407 | 0209 |
| 0808 | 0202 | 0505 | 0107 | 0111 | 0306 | 0506 | 0203 | 0909 |
| 0808 | 0202 | 0303 | 0107 | 1515 | 0408 | 0606 | 0303 | 0909 |
| 0808 | 0102 | 0507 | 0107 | 1515 | 0305 | 0607 | 0404 | 0608 |
| 0708 | 0102 | 0305 | 0202 | 0104 | 0306 | 0606 | 0308 | 0606 |
| 0808 | 0102 | 0303 | 0207 | 0113 | 0505 | 0507 | 0304 | 0608 |
| 0308 | 0206 | 0307 | 0101 | 0311 | 0408 | 0606 | 0404 | 0606 |
| 0808 | 0202 | 0307 | 0107 | 0111 | 0305 | 0606 | 0304 | 0608 |
| 0308 | 0204 | 0307 | 0107 | 1115 | 0308 | 0608 | 0304 | 0508 |
| 0303 | 0102 | 0505 | 0101 | 0101 | 0406 | 0608 | 0404 | 0808 |
| 0308 | 0102 | 0305 | 0407 | 0115 | 0303 | 0608 | 0404 | 0809 |
| 0708 | 0202 | 0303 | 0107 | 0415 | 0608 | 0506 | 0304 | 0809 |
| 0707 | 0202 | 0307 | 0101 | 0101 | 0303 | 0808 | 0708 | 0808 |
| 0307 | 0206 | 0305 | 0707 | 0315 | 0408 | 0606 | 0404 | 0606 |
| 0707 | 0202 | 0305 | 0707 | 0415 | 0308 | 0609 | 0308 | 0606 |
| 0808 | 0101 | 0505 | 0101 | 1515 | 0305 | 0607 | 0304 | 0808 |
| 0708 | 0202 | 0507 | 0101 | 1115 | 0508 | 0606 | 0404 | 0608 |
| 0808 | 0202 | 0507 | 0407 | 1115 | 0306 | 0606 | 0204 | 0809 |
| 0708 | 0202 | 0307 | 0101 | 1515 | 0808 | 0809 | 0304 | 0608 |
| 0808 | 0204 | 0707 | 0101 | 0101 | 0606 | 0609 | 0405 | 0606 |
| TAT |  |  |  |  |  |  |  |  |
| 0608 | 0202 | 0305 | 0607 | 0111 | 0307 | 0608 | 0405 | 0613 |
| 0606 | 0607 | 0507 | 0101 | 1315 | 0608 | 0406 | 0204 | 0608 |
| 0608 | 0202 | 0508 | 0101 | 0111 | 0406 | 0406 | 0304 | 0606 |
| 0708 | 0204 | 0307 | 0207 | 1515 | 0506 | 0607 | 0307 | 0613 |
| 0707 | 0102 | 0305 | 0107 | 0115 | 0506 | 0606 | 0304 | 0810 |
| 0708 | 0202 | 0308 | 0707 | 0101 | 0404 | 0609 | 0303 | 0910 |
| 0708 | 0204 | 0305 | 0707 | 0115 | 0408 | 0608 | 0405 | 0909 |
| 0708 | 0406 | 0305 | 0106 | 1315 | 0508 | 0506 | 0304 | 0609 |
| 0708 | 0206 | 0305 | 0207 | 1315 | 0306 | 0608 | 0205 | 0606 |
| 0708 | 0206 | 0305 | 0102 | 0106 | 0608 | 0606 | 0303 | 0606 |
| 0607 | 0202 | 0303 | 0101 | 0113 | 0505 | 0506 | 0203 | 0606 |
| 0708 | 0202 | 0303 | 0104 | 0101 | 0306 | 0606 | 0305 | 0606 |
| 0608 | 0102 | 0305 | 0107 | 0115 | 0407 | 0606 | 0304 | 0609 |
| 0608 | 0202 | 0305 | 0707 | 0115 | 0407 | 0608 | 0405 | 0913 |
| 0607 | 0102 | 0308 | 0404 | 0101 | 0404 | 0606 | 0406 | 0606 |
| 0608 | 0202 | 0507 | 0407 | 1515 | 0308 | 0606 | 0404 | 0209 |
| 0607 | 0202 | 0305 | 0107 | 1313 | 0304 | 0808 | 0204 | 0613 |
| 0708 | 0204 | 0307 | 0101 | 1115 | 0608 | 0606 | 0307 | 0208 |
| 0709 | 0202 | 0303 | 0101 | 0114 | 0606 | 0606 | 0203 | 0509 |
| 0708 | 0102 | 0307 | 0101 | 1515 | 0505 | 0606 | 0303 | 0608 |
| 0708 | 0202 | 0307 | 0707 | 0115 | 0606 | 0505 | 0407 | 0913 |
| 0707 | 0202 | 0308 | 0107 | 0115 | 0405 | 0609 | 0304 | 0909 |
| 0608 | 0202 | 0507 | 0707 | 1515 | 0305 | 0606 | 0304 | 0809 |
| 0708 | 0102 | 0506 | 0101 | 0111 | 0508 | 0506 | 0304 | 0608 |
| 0608 | 0202 | 0607 | 0101 | 0115 | 0406 | 0408 | 0303 | 0809 |
| 0606 | 0102 | 0708 | 0104 | 0101 | 0404 | 0606 | 0306 | 0609 |
| 0606 | 0102 | 0308 | 0101 | 0115 | 0304 | 0606 | 0306 | 0609 |
| 0808 | 0404 | 0506 | 0107 | 0115 | 0505 | 0709 | 0203 | 0606 |
| R-U |  |  |  |  |  |  |  |  |
| 0209 | 0106 | 0404 | 0607 | 0101 | 0304 | 0204 | 0304 | 0505 |
| 1010 | 0506 | 0606 | 0607 | 0206 | 0304 | 0404 | 0303 | 0405 |
| 0512 | 0101 | 0304 | 0707 | 0108 | 0406 | 0406 | 0506 | 0406 |
| 1011 | 0405 | 0303 | 0607 | 0808 | 0406 | 0405 | 0304 | 1114 |
| 0404 | 0505 | 0304 | 0607 | 0206 | 0304 | 0404 | 0404 | 1112 |
| 0509 | 0406 | 0303 | 0607 | 0101 | 0405 | 0405 | 0506 | 0406 |
| 0405 | 0105 | 0305 | 0708 | 0106 | 0404 | 0104 | 0206 | 0405 |
| 0510 | 0106 | 0204 | 0607 | 0606 | 0406 | 0406 | 0305 | 0405 |
| 0512 | 0101 | 0304 | 0707 | 0108 | 0406 | 0406 | 0506 | 0406 |
| 0512 | 0101 | 0304 | 0707 | 0606 | 0406 | 0406 | 0506 | 0406 |
| 0404 | 0505 | 0304 | 0607 | 0206 | 0304 | 0104 | 0404 | 0606 |
| 1011 | 0405 | 0405 | 0707 | 0106 | 0303 | 0204 | 0205 | 1012 |
| R-K |  |  |  |  |  |  |  |  |
| 0709 | 0505 | 0303 | 0707 | 0102 | 0304 | 0407 | 0204 | 0410 |
| 0614 | 0505 | 0305 | 0808 | 0106 | 0303 | 0405 | 0204 | 0408 |
| 0713 | 0206 | 0103 | 0607 | 0206 | 0306 | 0404 | 0508 | 0610 |
| 0909 | 0104 | 0306 | 0607 | 0207 | 0304 | 0405 | 0304 | 0410 |
| 0508 | 0305 | 0306 | 0607 | 0202 | 0306 | 0404 | 0405 | 0611 |
| 0412 | 0505 | 0406 | 0607 | 0107 | 0505 | 0404 | 0404 | 0104 |
| 0911 | 0405 | 0307 | 0707 | 0110 | 0303 | 0405 | 0305 | 0609 |
| 0910 | 0405 | 0305 | 0708 | 0106 | 0305 | 0405 | 0405 | 0610 |
| 0712 | 0505 | 0305 | 0708 | 0202 | 0505 | 0404 | 0404 | 1011 |
| 0509 | 0105 | 0305 | 0707 | 0606 | 0305 | 0404 | 0405 | 0610 |
| 0405 | 0505 | 0305 | 0708 | 0106 | 0306 | 0405 | 0505 | 0406 |
| 0912 | 0505 | 0306 | 0707 | 0206 | 0303 | 0404 | 0505 | 0910 |
| 1011 | 0405 | 0303 | 0708 | 0108 | 0303 | 0404 | 0404 | 0405 |
| 0909 | 0405 | 0306 | 0708 | 0107 | 0307 | 0405 | 0404 | 0110 |
| 0912 | 0405 | 0606 | 0607 | 0106 | 0303 | 0505 | 0405 | 0910 |
| 0505 | 0103 | 0506 | 0607 | 0507 | 0304 | 0406 | 0105 | 1013 |
| 1010 | 0405 | 0306 | 0607 | 0106 | 0304 | 0405 | 0505 | 0409 |
| 0513 | 0205 | 0305 | 0607 | 0610 | 0304 | 0404 | 0408 | 0611 |
| 0510 | 0405 | 0305 | 0607 | 0102 | 0304 | 0506 | 0103 | 0510 |
| 0409 | 0406 | 0303 | 0707 | 0102 | 0303 | 0507 | 0404 | 0410 |
| 0410 | 0505 | 0303 | 0808 | 0707 | 0304 | 0404 | 0304 | 0606 |
| 0509 | 0204 | 0303 | 0707 | 0607 | 0303 | 0406 | 0405 | 0209 |
| 0610 | 0505 | 0304 | 0607 | 0102 | 0304 | 0404 | 0404 | 1011 |
| 0911 | 0103 | 0303 | 0607 | 0707 | 0304 | 0405 | 0304 | 0610 |
| 0910 | 0505 | 0305 | 0607 | 0101 | 0303 | 0606 | 0305 | 0610 |
| 0909 | 0405 | 0305 | 0607 | 0507 | 0304 | 0404 | 0304 | 1011 |
| 1010 | 0505 | 0305 | 0707 | 0106 | 0307 | 0404 | 0304 | 1010 |
| 0606 | 0505 | 0103 | 0707 | 0102 | 0406 | 0404 | 0404 | 1111 |
| 1011 | 0405 | 0303 | 0707 | 0207 | 0506 | 0405 | 0304 | 0106 |
| 0512 | 0405 | 0305 | 0607 | 0208 | 0304 | 0707 | 0205 | 0410 |
| 0712 | 0104 | 0304 | 0607 | 0102 | 0303 | 0406 | 0405 | 0211 |
| 0610 | 0405 | 0304 | 0607 | 0208 | 0306 | 0404 | 0404 | 1115 |
| 0612 | 0405 | 0307 | 0707 | 0106 | 0303 | 0405 | 0404 | 1516 |
| SWE |  |  |  |  |  |  |  |  |
| 0509 | 0204 | 0308 | 0505 | 0108 | 0405 | 0506 | 0304 | 0909 |
| 0707 | 0202 | 0405 | 0606 | 0509 | 0206 | 0606 | 0507 | 0506 |
| 0507 | 0202 | 0808 | 0404 | 0111 | 0404 | 0607 | 0405 | 0505 |
| 0505 | 0204 | 0406 | 0808 | 0105 | 0405 | 0607 | 0406 | 0710 |
| 0308 | 0204 | 0307 | 0101 | 0108 | 0405 | 0606 | 0107 | 0912 |
| 0708 | 0404 | 0608 | 0707 | 0714 | 0406 | 0608 | 0405 | 0205 |
| 0508 | 0407 | 0303 | 0404 | 0109 | 0404 | 0606 | 0404 | 0310 |
| 0507 | 0405 | 0307 | 0101 | 0313 | 0404 | 0606 | 0303 | 0305 |
| 0608 | 0206 | 0306 | 0707 | 0205 | 0305 | 0606 | 0404 | 0611 |
| 0407 | 0204 | 0306 | 0505 | 0106 | 0204 | 0606 | 0506 | 0911 |
| 0407 | 0204 | 0304 | 0708 | 0111 | 0408 | 0607 | 0407 | 0609 |
| 0607 | 0202 | 0303 | 0101 | 0509 | 0404 | 0607 | 0205 | 0412 |
| 0607 | 0406 | 0505 | 0809 | 0109 | 0405 | 0606 | 0303 | 0306 |
| 0509 | 0202 | 0308 | 0808 | 0105 | 0404 | 0608 | 0304 | 0307 |
| LEZ |  |  |  |  |  |  |  |  |
| 0404 | 0404 | 0303 | 0108 | 0101 | 0505 | 0206 | 0202 | 0609 |
| 0407 | 0204 | 0307 | 0408 | 0101 | 0508 | 0607 | 0204 | 0609 |
| 0407 | 0204 | 0307 | 0104 | 0107 | 0506 | 0609 | 0405 | 0606 |
| 0407 | 0204 | 0307 | 0101 | 0107 | 0809 | 0609 | 0305 | 0609 |
| 0105 | 0404 | 0606 | 0101 | 0505 | 0909 | 0206 | 0205 | 0000 |
| 0808 | 0202 | 0305 | 0107 | 0103 | 0408 | 0609 | 0304 | 0611 |
| 0308 | 0204 | 0303 | 0107 | 0102 | 0304 | 0204 | 0303 | 1011 |
| 0404 | 0404 | 0306 | 0101 | 0105 | 0204 | 0606 | 0205 | 0909 |
| 0808 | 0202 | 0407 | 0104 | 0103 | 0408 | 0609 | 0304 | 0909 |
| 0404 | 0404 | 0306 | 0108 | 0107 | 0405 | 0606 | 0202 | 0909 |
| 0508 | 0205 | 0307 | 0104 | 0202 | 0808 | 0507 | 0304 | 0611 |
| 0406 | 0404 | 0304 | 0708 | 0101 | 0505 | 0506 | 0505 | 0909 |
| 0304 | 0204 | 0607 | 0408 | 0101 | 0509 | 0607 | 0203 | 0909 |
| 0404 | 0404 | 0306 | 0101 | 0105 | 0506 | 0606 | 0505 | 0606 |
| 0404 | 0404 | 0306 | 0808 | 0507 | 0406 | 0606 | 0205 | 0909 |
| 0404 | 0404 | 0103 | 0808 | 0105 | 0205 | 0606 | 0202 | 0909 |
| 0404 | 0404 | 0406 | 0808 | 0101 | 0205 | 0606 | 0205 | 0609 |
| 0404 | 0404 | 0306 | 0101 | 0107 | 0406 | 0606 | 0505 | 0909 |
| 0304 | 0404 | 0607 | 0808 | 0101 | 0108 | 0305 | 0206 | 0609 |
| 0104 | 0404 | 0606 | 0707 | 0101 | 0304 | 0606 | 0202 | 0909 |
| 0304 | 0204 | 0607 | 0808 | 0101 | 0505 | 0606 | 0203 | 0909 |
| 0208 | 0204 | 0307 | 0106 | 0105 | 0405 | 0609 | 0405 | 0609 |
| 0404 | 0404 | 0306 | 0101 | 0107 | 0406 | 0606 | 0505 | 0609 |
| 0408 | 0204 | 0307 | 0101 | 0305 | 0405 | 0606 | 0405 | 0609 |
| WIS |  |  |  |  |  |  |  |  |
| 0811 | 0507 | 0404 | 0101 | 0113 | 0306 | 0406 | 0203 | 0812 |
| 0708 | 0107 | 0304 | 0101 | 1315 | 0606 | 0608 | 0203 | 0708 |
| 0303 | 0202 | 0307 | 0407 | 0102 | 0408 | 0407 | 0303 | 0810 |
| 0307 | 0204 | 0507 | 0101 | 0101 | 0308 | 0607 | 0304 | 0809 |
| 0808 | 0206 | 0305 | 0207 | 0112 | 0306 | 0609 | 0304 | 0609 |
| 0404 | 0404 | 0505 | 0506 | 0105 | 0404 | 0407 | 0505 | 0404 |
| 0204 | 0404 | 0304 | 0606 | 0511 | 0203 | 0204 | 0203 | 1111 |
| 0808 | 0206 | 0307 | 0101 | 0000 | 0303 | 0606 | 0000 | 0609 |
| 0708 | 0204 | 0307 | 0106 | 0211 | 0308 | 0406 | 0204 | 0609 |
| 0405 | 0304 | 0303 | 0606 | 0206 | 0203 | 0404 | 0205 | 0304 |
| 0408 | 0202 | 0103 | 0107 | 0202 | 0408 | 0407 | 0303 | 0610 |
| 0308 | 0206 | 0307 | 0101 | 0105 | 0408 | 0407 | 0303 | 0610 |
| 0808 | 0206 | 0307 | 0101 | 0111 | 0308 | 0406 | 0303 | 0609 |
| 0708 | 0202 | 0305 | 0407 | 0103 | 0406 | 0509 | 0404 | 0506 |
| 0808 | 0101 | 0204 | 0306 | 0000 | 0000 | 0405 | 0303 | 0606 |
| 0305 | 0304 | 0406 | 0106 | 0205 | 0203 | 0404 | 0305 | 0810 |
| 0808 | 0102 | 0304 | 0101 | 0115 | 0606 | 0809 | 0304 | 0507 |
| 0811 | 0206 | 0507 | 0404 | 0102 | 0408 | 0405 | 0304 | 0506 |
| 0305 | 0205 | 0103 | 0107 | 0102 | 0308 | 0407 | 0303 | 0611 |
| 0411 | 0405 | 0407 | 0606 | 0102 | 0304 | 0304 | 0305 | 1011 |
| 0308 | 0206 | 0307 | 0104 | 0101 | 0308 | 0607 | 0303 | 0609 |

Table C. Microsatellite polymorphisms in the investigated populations. *N*  - number of unique genotypes, identified in non-invasive samples (see Table A for details) or number of sampled individuals (see Material and Methods); *A* ­– mean number of alleles per locus; *H_O_* – heterozygosity observed; *H*_E_ – heterozygosity expected; *HWE* – *P-*values for HWE exact test for heterozygote deficiency/excess (* - *P*<0.05; ns – non-significant (*P>*0.05)); *F*_IS_ – fixation index (* – significant after Bonferroni correction); *P*_(ID)_ by locus ― Probability of Identity for each locus; All loci loci *P*_(ID)_/*P*_(ID-Sibs)_ – Probability of Identity for combination of 9 loci/Probability of Identity for combination of 9 loci, taking into account the genetic similarity among siblings

| **Locus** | ***A*** | ***H*_O_** | ***H*_E_** | ***HWE*** | ***F*_IS_** | ***P*_(ID)_ by locus** |
| --- | --- | --- | --- | --- | --- | --- |
|  | PA (*N* = 29) | | | | |  |
| **Bg16** | 6 | 0.759 | 0.698 | ns. | -0.069 | 0.138 |
| **TTT1** | 5 | 0.414 | 0.473 | ns. | 0.143 | 0.305 |
| **TUT2** | 6 | 0.793 | 0.755 | ns. | -0.033 | 0.101 |
| **Bg12** | 4 | 0.571 | 0.637 | ns. | 0.121 | 0.192 |
| **TUD4** | 7 | 0.655 | 0.801 | * | 0.199 | 0.065 |
| **TUT4** | 6 | 0.793 | 0.725 | ns. | -0.077 | 0.123 |
| **TUT3** | 5 | 0.690 | 0.651 | ns. | -0.042 | 0.182 |
| **Bg18** | 6 | 0.448 | 0.429 | ns. | -0.027 | 0.349 |
| **TUD5** | 7 | 0.828 | 0.795 | ns. | -0.024 | 0.074 |
| **All loci *P*_(ID)_/*P*_(ID-Sibs)_** |  |  |  |  |  | <0.001/0.001 |
|  | LUB (*N* = 20) | | | | |  |
| **Bg16** | 6 | 0.700 | 0.661 | ns. | -0.033 | 0.163 |
| **TTT1** | 2 | 0.200 | 0.255 | ns. | 0.240 | 0.588 |
| **TUT2** | 4 | 0.700 | 0.578 | ns. | -0.188 | 0.242 |
| **Bg12** | 3 | 0.526 | 0.630 | ns. | 0.191 | 0.214 |
| **TUD4** | 4 | 0.700 | 0.706 | ns. | 0.034 | 0.135 |
| **TUT4** | 3 | 0.600 | 0.521 | ns. | -0.126 | 0.341 |
| **TUT3** | 3 | 0.300 | 0.265 | ns. | -0.107 | 0.558 |
| **Bg18** | 4 | 0.600 | 0.554 | ns. | -0.058 | 0.244 |
| **TUD5** | 4 | 0.750 | 0.663 | ns. | -0.107 | 0.181 |
| **All loci *P*_(ID)_/*P*_(ID-Sibs)_** |  |  |  |  |  | <0.001/0.004 |
|  | GOR (*N* = 44) | | | | |  |
| **Bg16** | 3 | 0.523 | 0.592 | ns. | 0.128 | 0.253 |
| **TTT1** | 4 | 0.477 | 0.486 | ns. | 0.030 | 0.296 |
| **TUT2** | 4 | 0.750 | 0.624 | ns. | -0.190 | 0.216 |
| **Bg12** | 3 | 0.409 | 0.595 | * | 0.323 | 0.233 |
| **TUD4** | 4 | 0.591 | 0.543 | ns. | -0.077 | 0.291 |
| **TUT4** | 6 | 0.727 | 0.701 | ns. | -0.026 | 0.142 |
| **TUT3** | 4 | 0.636 | 0.566 | ns. | -0.112 | 0.242 |
| **Bg18** | 3 | 0.705 | 0.614 | * | -0.135 | 0.230 |
| **TUD5** | 4 | 0.523 | 0.524 | ns. | 0.013 | 0.286 |
| **All loci *P*_(ID)_/*P*_(ID-Sibs)_** |  |  |  |  |  | <0.001/0.003 |
|  | BPN (*N* = 35) | | | | |  |
| **Bg16** | 3 | 0.543 | 0.540 | ns. | 0.009 | 0.285 |
| **TTT1** | 4 | 0.400 | 0.378 | ns. | -0.045 | 0.420 |
| **TUT2** | 4 | 0.543 | 0.616 | ns. | 0.132 | 0.212 |
| **Bg12** | 4 | 0.400 | 0.652 | * | 0.399* | 0.189 |
| **TUD4** | 6 | 0.686 | 0.692 | ns. | 0.023 | 0.149 |
| **TUT4** | 5 | 0.771 | 0.753 | ns. | -0.009 | 0.100 |
| **TUT3** | 5 | 0.629 | 0.580 | ns. | -0.070 | 0.210 |
| **Bg18** | 6 | 0.629 | 0.656 | ns. | 0.056 | 0.182 |
| **TUD5** | 6 | 0.600 | 0.679 | ns. | 0.131 | 0.163 |
| **All loci *P*_(ID)_/*P*_(ID-Sibs)_** |  |  |  |  |  | <0.001/0.002 |
|  | TAT (*N* = 28) | | | | |  |
| **Bg16** | 4 | 0.786 | 0.675 | ns. | -0.146 | 0.175 |
| **TTT1** | 5 | 0.500 | 0.507 | ns. | 0.032 | 0.274 |
| **TUT2** | 5 | 0.893 | 0.719 | ns. | -0.224 | 0.123 |
| **Bg12** | 5 | 0.464 | 0.640 | ns. | 0.292 | 0.191 |
| **TUD4** | 6 | 0.679 | 0.673 | ns. | 0.011 | 0.165 |
| **TUT4** | 6 | 0.714 | 0.804 | ns. | 0.129 | 0.068 |
| **TUT3** | 6 | 0.500 | 0.556 | ns. | 0.119 | 0.223 |
| **Bg18** | 6 | 0.821 | 0.724 | ns. | -0.116 | 0.116 |
| **TUD5** | 7 | 0.679 | 0.718 | ns. | 0.073 | 0.120 |
| **All loci *P*_(ID)_/*P*_(ID-Sibs)_** |  |  |  |  |  | <0.001/0.001 |
|  | R-U (*N* = 12) | | | | |  |
| **Bg16** | 7 | 0.750 | 0.819 | * | 0.128 | 0.057 |
| **TTT1** | 4 | 0.583 | 0.705 | ns. | 0.214 | 0.141 |
| **TUT2** | 5 | 0.667 | 0.670 | ns. | 0.049 | 0.168 |
| **Bg12** | 3 | 0.667 | 0.469 | ns. | -0.386 | 0.360 |
| **TUD4** | 4 | 0.583 | 0.705 | ns. | 0.214 | 0.141 |
| **TUT4** | 4 | 0.833 | 0.642 | ns. | -0.257 | 0.188 |
| **TUT3** | 5 | 0.833 | 0.611 | ns. | -0.325 | 0.186 |
| **Bg18** | 5 | 0.750 | 0.781 | * | 0.083 | 0.084 |
| **TUD5** | 7 | 0.833 | 0.792 | ns. | -0.009 | 0.074 |
| **All loci *P*_(ID)_/*P*_(ID-Sibs)_** |  |  |  |  |  | <0.001/0.001 |
|  | R-K (*N* = 33) | | | | |  |
| **Bg16** | 11 | 0.788 | 0.858 | ns. | 0.097 | 0.035 |
| **TTT1** | 6 | 0.667 | 0.616 | * | -0.067 | 0.192 |
| **TUT2** | 6 | 0.758 | 0.629 | ns. | -0.190 | 0.176 |
| **Bg12** | 3 | 0.636 | 0.537 | * | -0.171 | 0.277 |
| **TUD4** | 7 | 0.818 | 0.788 | ns. | -0.023 | 0.077 |
| **TUT4** | 5 | 0.636 | 0.597 | ns. | -0.050 | 0.201 |
| **TUT3** | 4 | 0.485 | 0.538 | ns. | 0.114 | 0.261 |
| **Bg18** | 6 | 0.636 | 0.661 | ns. | 0.053 | 0.162 |
| **TUD5** | 12 | 0.909 | 0.836 | ns. | -0.073 | 0.045 |
| **All loci *P*_(ID)_/*P*_(ID-Sibs)_** |  |  |  |  |  | <0.001/0.001 |
|  | SWE (*N* = 14) | | | | |  |
| **Bg16** | 7 | 0.857 | 0.791 | ns. | -0.047 | 0.072 |
| **TTT1** | 5 | 0.643 | 0.615 | ns. | -0.009 | 0.218 |
| **TUT2** | 6 | 0.714 | 0.765 | ns. | 0.103 | 0.085 |
| **Bg12** | 7 | 0.786 | 0.832 | ns. | 0.092 | 0.052 |
| **TUD4** | 11 | 1.000 | 0.827 | ns. | -0.174 | 0.048 |
| **TUT4** | 6 | 0.643 | 0.587 | ns. | -0.059 | 0.205 |
| **TUT3** | 4 | 0.500 | 0.411 | ns. | -0.182 | 0.378 |
| **Bg18** | 7 | 0.714 | 0.776 | ns. | 0.116 | 0.081 |
| **TUD5** | 10 | 0.857 | 0.872 | ns. | 0.055 | 0.030 |
| **All loci *P*_(ID)_/*P*_(ID-Sibs)_** |  |  |  |  |  | <0.001/<0.001 |
|  | LEZ (*N* = 24) | | | | |  |
| **Bg16** | 8 | 0.542 | 0.641 | ns. | 0.175 | 0.157 |
| **TTT1** | 3 | 0.375 | 0.424 | ns. | 0.138 | 0.405 |
| **TUT2** | 6 | 0.833 | 0.710 | ns. | -0.153 | 0.134 |
| **Bg12** | 5 | 0.458 | 0.670 | ns. | 0.335 | 0.164 |
| **TUD4** | 5 | 0.583 | 0.609 | ns. | 0.063 | 0.190 |
| **TUT4** | 8 | 0.792 | 0.805 | ns. | 0.037 | 0.063 |
| **TUT3** | 7 | 0.542 | 0.532 | ns. | 0.003 | 0.240 |
| **Bg18** | 5 | 0.625 | 0.728 | ns. | 0.163 | 0.122 |
| **TUD5** | 4 | 0.478 | 0.532 | * | 0.123 | 0.292 |
| **All loci *P*_(ID)_/*P*_(ID-Sibs)_** |  |  |  |  |  | <0.001/0.001 |
|  | WIS (*N* = 21) | | | | |  |
| **Bg16** | 7 | 0.667 | 0.748 | ns. | 0.133 | 0.092 |
| **TTT1** | 7 | 0.714 | 0.770 | * | 0.096 | 0.082 |
| **TUT2** | 7 | 0.857 | 0.766 | ns. | -0.094 | 0.089 |
| **Bg12** | 7 | 0.476 | 0.709 | * | 0.350 | 0.124 |
| **TUD4** | 9 | 0.842 | 0.763 | ns. | -0.077 | 0.085 |
| **TUT4** | 5 | 0.800 | 0.768 | ns. | -0.017 | 0.092 |
| **TUT3** | 8 | 0.857 | 0.768 | ns. | -0.093 | 0.084 |
| **Bg18** | 4 | 0.550 | 0.608 | ns. | 0.120 | 0.197 |
| **TUD5** | 10 | 0.857 | 0.847 | * | 0.012 | 0.039 |
| **All loci *P*_(ID)_/*P*_(ID-Sibs)_** |  |  |  |  |  | <0.001/<0.001 |
|  | All Polish natural populations (*N* = 156) | | | | |  |
| **Bg16** | 9 | 0.641 | 0.711 | * | 0.102 | 0.128 |
| **TTT1** | 6 | 0.417 | 0.445 | ns. | 0.067 | 0.330 |
| **TUT2** | 7 | 0.731 | 0.704 | ns. | -0.034 | 0.131 |
| **Bg12** | 6 | 0.461 | 0.739 | * | 0.379* | 0.114 |
| **TUD4** | 13 | 0.654 | 0.734 | * | 0.113 | 0.101 |
| **TUT4** | 7 | 0.731 | 0.783 | ns. | 0.070 | 0.080 |
| **TUT3** | 6 | 0.577 | 0.655 | ns. | 0.123 | 0.165 |
| **Bg18** | 8 | 0.647 | 0.723 | * | 0.108 | 0.117 |
| **TUD5** | 10 | 0.654 | 0.782 | * | 0.167* | 0.071 |
| **All loci *P*_(ID)_/*P*_(ID-Sibs)_** |  |  |  |  |  | <0.001/0.001 |
|  | All natural populations (*N* = 215) | | | | |  |
| **Bg16** | 13 | 0.684 | 0.808 | * | 0.156* | 0.060 |
| **TTT1** | 7 | 0.479 | 0.633 | * | 0.246* | 0.163 |
| **TUT2** | 8 | 0.730 | 0.712 | ns. | -0.024 | 0.119 |
| **Bg12** | 8 | 0.521 | 0.775 | * | 0.329* | 0.085 |
| **TUD4** | 15 | 0.698 | 0.788 | * | 0.117* | 0.063 |
| **TUT4** | 7 | 0.716 | 0.785 | ns. | 0.090 | 0.080 |
| **TUT3** | 8 | 0.572 | 0.720 | * | 0.208* | 0.115 |
| **Bg18** | 8 | 0.656 | 0.748 | * | 0.125* | 0.102 |
| **TUD5** | 16 | 0.716 | 0.830 | * | 0.139* | 0.045 |
| **All loci *P*_(ID)_/*P*_(ID-Sibs)_** |  |  |  |  |  | <0.001/<0.001 |
|  | All populations (*N* = 260) | | | | |  |
| **Bg16** | 14 | 0.669 | 0.822 | * | 0.187* | 0.052 |
| **TTT1** | 7 | 0.488 | 0.666 | * | 0.269* | 0.144 |
| **TUT2** | 8 | 0.750 | 0.728 | * | -0.029 | 0.107 |
| **Bg12** | 9 | 0.512 | 0.785 | * | 0.350* | 0.078 |
| **TUD4** | 16 | 0.698 | 0.782 | * | 0.110* | 0.064 |
| **TUT4** | 9 | 0.730 | 0.799 | * | 0.088 | 0.071 |
| **TUT3** | 9 | 0.592 | 0.727 | * | 0.187* | 0.108 |
| **Bg18** | 8 | 0.645 | 0.753 | * | 0.146* | 0.099 |
| **TUD5** | 16 | 0.707 | 0.828 | * | 0.148* | 0.047 |
| **All loci *P*_(ID)_/*P*_(ID-Sibs)_** |  |  |  |  |  | <0.001/<0.001 |

Table D. Genetic differentiation among 8 natural populations and 2 breeding flocks (*n* = 260) of the capercaillie, estimated using standardized measures: *D*_EST_ and *F*'_ST_

|  | PA | LUB | GOR | BPN | TAT | R-U | R-K | SWE | LEZ | WIS |
| --- | --- | --- | --- | --- | --- | --- | --- | --- | --- | --- |
| PA |  | 0.38 | 0.26 | 0.37 | 0.30 | 0.50 | 0.51 | 0.27 | 0.45 | 0.28 |
| LUB | 0.49 |  | 0.45 | 0.49 | 0.44 | 0.61 | 0.60 | 0.41 | 0.40 | 0.41 |
| GOR | 0.34 | 0.59 |  | 0.13 | 0.09 | 0.56 | 0.56 | 0.28 | 0.38 | 0.29 |
| BPN | 0.43 | 0.59 | 0.17 |  | 0.05 | 0.56 | 0.51 | 0.30 | 0.37 | 0.19 |
| TAT | 0.36 | 0.56 | 0.14 | 0.06 |  | 0.49 | 0.50 | 0.18 | 0.28 | 0.18 |
| R-U | 0.64 | 0.74 | 0.67 | 0.67 | 0.60 |  | 0.14 | 0.36 | 0.51 | 0.33 |
| R-K | 0.67 | 0.74 | 0.67 | 0.63 | 0.61 | 0.17 |  | 0.41 | 0.54 | 0.35 |
| SWE | 0.33 | 0.56 | 0.35 | 0.35 | 0.21 | 0.48 | 0.56 |  | 0.23 | 0.23 |
| LEZ | 0.57 | 0.59 | 0.51 | 0.49 | 0.38 | 0.65 | 0.65 | 0.27 |  | 0.31 |
| WIS | 0.35 | 0.58 | 0.39 | 0.27 | 0.25 | 0.42 | 0.46 | 0.34 | 0.41 |  |

Above diagonal - *D*_EST_ (i), below diagonal - *F'*_ST_ (ii). All *D*_EST_ values are significant after Bonferroni correction (1000 randomizations, adjusted P-value = 0.045). Overall *D*_EST_ = 0.376 (*P* < 0.001).
